# Clinical Frailty Scale (CFS) indicated frailty is associated with increased in-hospital and 30-day mortality in COVID-19 patients: a systematic review and meta-analysis

**DOI:** 10.1186/s13613-021-00977-4

**Published:** 2022-02-20

**Authors:** Máté Rottler, Klementina Ocskay, Zoltán Sipos, Anikó Görbe, Marcell Virág, Péter Hegyi, Tihamér Molnár, Bálint Erőss, Tamás Leiner, Zsolt Molnár

**Affiliations:** 1 Institute for Translational Medicine, Szentágothai Research Centre, Medical School, University of Pécs, Pécs, Hungary; 2 Centre for Translational Medicine, Semmelweis University, Budapest, Hungary; 3 Division of Pancreatic Diseases, Heart and Vascular Center, Semmelweis University, Budapest, Hungary; 4 Department of Anaesthesiology and Intensive Therapy, Szent György University Teaching Hospital of Fejér County, 8000 Székesfehérvár, Hungary; 5 Doctoral School of Clinical Medicine, University of Szeged, 6720 Szeged, Hungary; 6 Department of Anaesthesiology and Intensive Therapy, University of Pécs, 7624 Pécs, Hungary; 7 Anaesthetic Department, Hinchingbrooke Hospital, North West Anglia NHS Foundation Trust, Huntingdon, PE29 6NT UK; 8 Department of Anaesthesiology and Intensive Therapy, Poznan University of Medical Sciences, 61-701 Poznan, Poland; 9 Department of Anaesthesiology and Intensive Therapy, Semmelweis University, 1082 Budapest, Hungary

**Keywords:** Intensive care, Clinical Frailty Scale, Hospital Frailty Risk Score, Ceiling of care, Geriatric

## Abstract

**Background:**

The concept of frailty provides an age-independent, easy-to-use tool for risk stratification. We aimed to summarize the evidence on the efficacy of frailty tools in risk assessment in COVID-19 patients.

**Methods:**

The protocol was registered (CRD42021241544). Studies reporting on frailty in COVID-19 patients were eligible. The main outcomes were mortality, length of hospital stay (LOH) and intensive care unit (ICU) admission in frail and non-frail COVID-19 patients. Frailty was also compared in survivors and non-survivors. Five databases were searched up to 24th September 2021. The QUIPS tool was used for the risk of bias assessment. Odds ratios (OR) and weighted mean differences (WMD) were calculated with 95% confidence intervals (CI) using a random effect model. Heterogeneity was assessed using the *I*^2^ and *χ*^2^ tests.

**Results:**

From 3640 records identified, 54 were included in the qualitative and 42 in the quantitative synthesis. Clinical Frailty Scale (CFS) was used in 46 studies, the Hospital Frailty Risk Score (HFRS) by 4, the Multidimensional Prognostic Index (MPI) by 3 and three studies used other scores. We found that patients with frailty (CFS 4–9 or HFRS ≥ 5) have a higher risk of mortality (CFS: OR: 3.12; CI 2.56–3.81; HFRS OR: 1.98; CI 1.89–2.07). Patients with frailty (CFS 4–9) were less likely to be admitted to ICU (OR 0.28, CI 0.12–0.64). Quantitative synthesis for LOH was not feasible. Most studies carried a high risk of bias.

**Conclusions:**

As determined by CFS, frailty is strongly associated with mortality; hence, frailty-based patient management should be included in international COVID-19 treatment guidelines. Future studies investigating the role of frailty assessment on deciding ICU admission are strongly warranted.

**Supplementary Information:**

The online version contains supplementary material available at 10.1186/s13613-021-00977-4.

## Background

Almost 2 years after identifying the first SARS-CoV-2-infected patient, health care systems around the world periodically still face significant challenges; therefore, identifying factors that predict negative outcomes in COVID-19 is essential. The use of risk stratification tools for protocolized admission and determination of ceiling of care could help the decision-making and create transparency in these uncertain times.

Frailty describes a state of reduced physical, physiologic and cognitive reserve as a consequence of an ongoing accumulation of various deficits through time leading to increased vulnerability to stressors [1]. Although frailty is linked to ageing, progression in every individual is distinct. Nevertheless, frailty has been shown to be an age independent risk factor of mortality especially in the elderly population. Apparently, there is an urge to measure frailty within the scope of a risk stratification tool. However, the wide variety of frailty tools makes frailty assessment heterogenous. The Clinical Frailty Scale (CFS) was created by Rockwood et al. in 2005 to provide a simple approach with good predictive value [2]. The original 7-point scale was later upgraded to 9-points, one for the “severely frail”, “very severely frail”, and one for the “terminally ill” [3]. In the terminology used until 2020, points 1 to 4 covered patients described as “very fit”, “well”, “managing well”, and “vulnerable”. The revision published by Rockwood and Theou classified formerly “well” patients to “fit” and “vulnerable” patients to “living with very mild frailty” [4]. The score assesses different levels of functional independence, hence integrating a progressive accumulation of morbidity, loss of physical and cognitive function in a joint phenotype. It is meant to reflect a baseline health state 2 weeks before the onset of an acute condition [5].

The CFS is widely used in different clinical settings [6]. CFS outperformed the Charlson comorbidity index and age in predicting in-hospital mortality of patients older than 75 years with emergency hospital admission [7] and is an independent predictor of short- and long-term mortality in patients over 70 admitted to the ICU [8].

The Hospital Frailty Risk Score (HFRS) was developed to assess risk of frailty in older individuals automatically from routinely collected data using the International Classification of Diseases version 10 (ICD-10) codes [9]. On the one hand it is a useful tool for research and sociodemographic observations, on the other hand it has a potential for implementation into an automated hospital electronic system for acute clinical risk stratification [9].

The Multidimensional Prognostic Index (MPI) was developed as a prognostic tool for elderly patients. It is mainly used in the geriatrics as a part of a comprehensive geriatric assessment process. It has been validated for short-term and long-term outcomes alike and is not only meant as a tool for risk stratification, but also to enhance geriatric care by aiding to target specific interventions, thus improving outcomes [10]. Although it is a valuable tool in geriatric management, but its usefulness is limited in other fields and especially in critical care.

Frailty assessment was adopted in many guidelines in the triage of COVID-19 patients to aid decision-making regarding intensive care admission or the commencement of mechanical ventilation [11]. Recent studies and a meta-analyses reported higher odds and hazard ratios for mortality in frail COVID-19 patients [12–19].

We aimed to provide a detailed summary on the use of frailty tools in COVID-19, assessing the odds of patients with frailty for in-hospital and 30-day mortality, ICU admission, and length of hospitalization (LOH).

## Methods

### Protocol registration and reporting

The protocol was prospectively registered via PROSPERO under Registration number CRD42021241544. There was no deviation from the protocol. We report our results following the Preferred Reporting Items for Systematic Reviews and Meta-Analyses (PRISMA) recommendations [20] (Additional file 2).

### Eligibility and definitions

We formulated our clinical question using the PECO format. Based on preliminary searches, we chose to use two PECOs. We selected studies reporting on adult hospitalized patients with COVID-19, comparing frail (or frailer) patients to not frail (or less frail) patients. The assessed outcomes were all-cause in-hospital and 30-day mortality, ICU admission, and LOH. In our other analysis, the average frailty score of deceased COVID-19 patients was compared to survivors’.

COVID-19 positivity was defined as clinical, radiological, or laboratory diagnosis [21]. Any validated frailty scores and indexes were included, as well as non-validated ones, if the record contained sufficient information on the used index.

Studies with original data reporting on at least ten patients were eligible independently of study design. Abstracts and full-texts were both accepted.

### Search and selection

We searched MEDLINE (via PubMed), EMBASE, Scopus, The Cochrane Central Register of Controlled Trials (CENTRAL), and Web of Science on the 24th of September 2021 for eligible articles. We used “Title, Abstract, Keywords” filter in Scopus. No other filters or restrictions were applied. We also scanned the reference lists of the included studies and their citations in Google Scholar. The following search key was used: (“covid 19” OR “Wuhan virus” OR coronavirus OR “2019 nCoV” OR SARS-cov-2) AND frail*.

After removing duplicates using a reference management software (EndNote X9, Clarivate Analytics), two review authors (MR and TL) independently screened titles, abstracts, and then full‐texts against predefined eligibility criteria. Discrepancies were resolved by a third review author (ZM). Inter-rater reliability was determined at every phase by Cohen’s kappa coefficient, where values 0.01–0.20 indicate slight, 0.21–0.40 indicate fair, 0.41–0.60 indicate moderate, 0.61–0.80 indicate substantial and 0.81–1.00 indicate almost perfect or perfect agreement, respectively [22].

Outcomes reported by at least three studies using the same frailty score comparing identical frailty subgroups were included in the meta-analysis. All other eligible studies were incorporated into the qualitative synthesis.

### Data collection

Data on the first author, publication year, countries, study design, number of patients in each comparison group, their baseline characteristics (sex, age), type of frailty score used, method of frailty assessment, training of the assessor and available primary and secondary outcome parameters were extracted by two independent review authors (MR and LT) in duplicate using our standardized data collection form in Microsoft Excel. Disagreements were resolved by a third independent investigator (KO). Data from studies reporting individual patient data or raw data were regrouped if statistically feasible. Overlapping populations were identified, and the study with the largest sample size was included in the analyses.

### Risk of bias

Following the recommendations of the Cochrane Collaboration, the Quality in Prognosis Studies (QUIPS) tool was used by MR and TL independently [23]. Disagreements were resolved by ZM. In the study participation domain, gender, age, ethnicity, and comorbidities were taken into account. Study attrition was not judged for retrospective studies. In the prognostic factor measurement domain, the specification of the frailty assessor, information about their training, and missing data on frailty were considered. Less than 10% missing data were considered low risk, 10–20% some concerns, and more than 20% resulted in high risk for the whole domain. Outcome measurement and statistical analysis domains carried low risk in most cases because mortality is an objective outcome, and we mostly used crude numbers of patients reported by the authors. In the case of ICU admission, a detailed protocol for ICU admission was needed. In the study confounding domain, studies separately reporting baseline information for the frailty groups were judged low risk if no clinically significant differences were seen, some concerns if some differences were seen, and high risk if no data was reported. The overall risk of bias was calculated using the suggestions of Grooten et al. [24].

### Statistical analysis

Our primary aim was to investigate the differences between the two groups (Frail group vs Not frail group). We only included studies using the same cutoff in each analysis; therefore, multiple analyses were performed with slightly different frailty cutoffs. Most eligible studies used arbitrary categorization and grouping of patients by frailty, therefore we ought to perform multiple analyses (e.g. CFS 1–3 vs 4–9; 1–4 vs 5–9; 1–5 vs 6–9).

For dichotomous outcomes, odds ratios (ORs) with their 95% confidence intervals (CI) were calculated from the original raw data of the articles. In some cases, crude ORs were extracted and pooled with the calculated ORs. For continuous outcomes, weighted mean differences (WMDs) with 95% CI were calculated from the original raw data of the articles except in some cases when standard deviations (SDs) and means were calculated from the first quartile, median, the third quartile, and sample size according to Wan’s method [25].

We used the random effect model by DerSimonian and Laird [26]. We estimated the heterogeneity using the *χ*^2^ test with a significance of *p* < 0.1 and the *I*^2^ indicator. We followed the Cochrane Handbook’s recommendations when interpreting heterogeneity (http://handbook.cochrane.org, Chapter 10), meaning that *I*^2^ values between 30 and 60% were considered as moderate heterogeneity, between 50 and 90% as substantial heterogeneity and as considerable heterogeneity above 75%. Results of each meta-analysis were displayed graphically using forest plots.

Subgroup analyses were performed in the analyses of mortality associated with CFS, where the subgroups were determined by country (United Kingdom; UK and non-UK), by age (older than 65 years and no age restriction), and by mortality (in-hospital mortality and 30-day mortality). In the case of ICU admission CFS 1–3 vs 4–9 we performed a subgroup analysis, where groups were determined by frailty-based decision making.

To determine the robustness of an assessment, we performed the leave-one-out sensitivity analysis for all outcomes when reasonable. Using this method, we could examine whether altering any assumptions may lead to different final interpretations or conclusions [27]. The potential for a “small study effect”, including publication bias, was examined by visual inspection of funnel plots. Furthermore, Egger’s test was performed for analyses including at least ten studies to indicate significant asymmetry by using a significance of *p* < 0.05.

All data management and statistical analyses were performed with Stata (version 16.0, StataCorp).

## Results

### Selection and characteristics of included studies

**Fig. 1:**
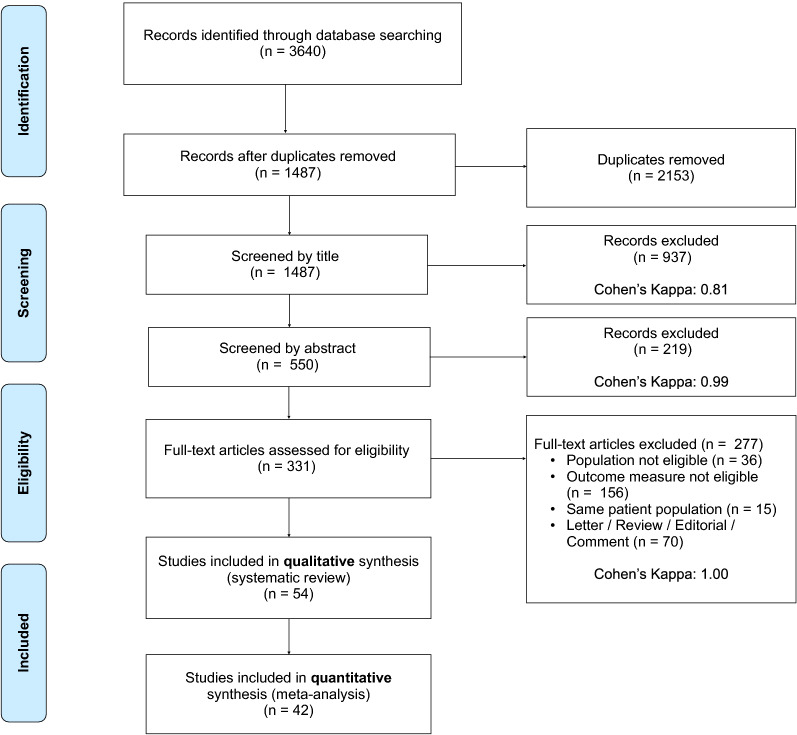
PRISMA flowchart of selection

The systematic search yielded 3640 records. After duplicate removal, 1487 records were screened by title, 550 by abstract, and 331 by full text. 54 studies were included in the qualitative and 42 in the quantitative synthesis. The detailed selection process and Cohen’s kappa values are shown in Fig. 1.

The most important aspects of each included study are presented in Table 1. Only cohort studies were enrolled. From the 54 studies, 10 collected data prospectively, 46 used the CFS, two the HFRS, two both, three the MPI, two studies a modified frailty index (mFI), and one the Frail Non-Disabled (FiND) questionnaire. All studies included patients from a clinical setting. Most studies enrolled patients over 65 years.

**Table 1:** Characteristics of included studies

Study	Origin	Recruitment period	Study type	Frailty Scale	Frailty assessment	Inclusion criteria		No. of patients		Age (years)		Sex	Outcomes
						Age (years)	COVID-19 diagnosis	Total	Deceased	Mean/Median	SD/IQ1–3	Male *n* (%)	
**Aliberti et al. **[28]	Brazil	30.03.2020–07.07.2020	R/C	CFS	R/P+	> 50y	PCR	1830	666	66	59–74	1061 (38)	30-day mortality
**Andrés-Esteban et al.** [29]	Spain	15.07.2020–31.07.2020	R/C	CFS	R/P+	0–97	PCR	254	104	70.16	16.01	155 (61)	In-hospital mortality; frailty diff. for in-hospital mort.; ICU admission; LOH
**Apea** [30]	UK	01.01.2020–13.05.2020	P/C	CFS, HFRS	R	> 16	PCR	831	315	n/a	n/a	n/a	30-day mortality
**Aw** [31]	UK	01.03.2020–30.04.2020	R/C	CFS	R/P+	> 65	PCR or Clin or Rad	677	271	81.1	8.1	366 (54.1)	In-hospital mortality, ICU admission
**Baker et al.** [32]	UK	31.01.2020–16.04.2020	R/C	CFS	R	> 18y	PCR	316	84	75	60–83	173 (54.7)	Frailty diff. for 30-day mort
**Bavaro et al.** [33]	Italy	01.03.2020–15.06.2020	R/C	CFS	R/P+	> 65y	PCR	206	56	80	72–86	98 (48)	In-hospital mortality; LOH
**Bielza **[34]	Spain	20.03.2020–01.06.2020	R/C	CFS	P+	> 70	PCR or Clin	630	282	87	82.9–91.1	223 (35.4)	30-day mortality, frailty diff. for 30-day mort. and severe vs non-severe cases
**Blomaard** [35]	Netherlands	27.02.2020–14.05.2020	R/C	CFS	R/P+	> 70	PCR or Clin or Rad	1376	499	78	74–84	830 (60.4)	In-hospital mortality, ICU admission, LOH, invasive ventilation, delirium, discharge destination
**Bradley** [36]	UK	01.04.2020–14.04.2020	P/C	CFS	P	n/r	PCR	830	300	70	58–80	509 (61.3)	30 day mortality, frailty diff. for 30-day mortality and 72 h mortality
Brill [37]	UK	10.03.2020–08.04.2020	R/C	CFS	P	n/r	PCR	450	173	72	56–83	272 (60)	Frailty diff. for in-hospital mortality
**Burns** [38]	UK	13.03.2020–22.04.2020	R/C	CFS	P	n/r	PCR	28	14	81.5	54–91	15 (54)	In-hospital mortality; frailty diff. for in-hospital mortality and duration of respiratory support
Cecchini et al. [39]	Italy	01.03.2020–30.04.2020	R/C	CFS	R	> 65y	PCR/CXR	122		87.1	6	55 (45.1)	Frailty diff. for in-hospital mort
**Chinnadurai **[40]	UK	13.03.2020–30.04.2020	R/C	CFS	P	n/r	PCR	215	86	74	60–82	133 (61.9)	In-hospital mortality
**Covino** [41]	Italy	01.0.4.2020–30.03.2021	P/C	CFS	P	> 80y	PCR	729	287	85	82–89	345 (47.3)	In-hospital mortality
Cuvelier [42]	Switzerland	13.03.2020–11.05.2020	R/C	CFS	R	> 80y	NR	20	10	87,1	82.8–90.6	14 (70)	Frailty diff. for in-hospital mort
**Davis **[43]	UK	18.03.2020–20.04.2020	R/C	CFS	R	n/r	PCR	222	95	82	56–99	74 (33)	30-day mortality
**De Smet** [44]	Belgium	12.03.2020–30.04.2020	R/C	CFS	P+	n/r	PCR	81	19	85	81–90	33 (41)	In-hospital mortality, frailty diff. for in-hospital mortality
**Dres et al.** [45]	France, Switzerland, Belgium	25.02.2020–04.05.2020	P/C	CFS	P	> 70y	PCR	1199	442	74	71–77	873 (72.8)	30-day mortality
**Fagard** [46]	Belgium	16.03.2020–16.05.2020	R/C	CFS	P+	> 70	PCR or Clin and CT	105	14	82	76–87	55 (52.4)	In-hospital mortality, frailty diff. for in-hospital mortality
Fallon et al. [47]	Ireland	25.03.2020–24.04.2020	R/C	CFS	R/P+	> 65y	PCR	86	29	77		n/r	Frailty diff. for 30-day mort
Fumagalli et al. [48]	Italy	22.02.2020–30.05.2020	R/C	mFI	R	> 75y	Clin	221	97	82	78–86	134 (60.6)	In-hospital mortality
**Gilis **[49]	France	03.03.2020–25.04.2020	P/C	CFS	P	> 75	PCR	186	56	85.3	5.78	92 (49.5)	30-day mortality, ICU admission, laboratory findings, symptoms, delirium, treatment
**Hewitt** [50]	UK, Italy	27.02.2020–28.04.2020	P/C	CFS	P+	> 18	PCR or Clin	1564	425	74	61–83	903 (57.7)	In-hospital mortality; LOH
Hoek [51]	Netherland	27.02.2020–30.04.2020	R/C	CFS	R	n/r	PCR	23	5	n/a	n/a	18 (78.3)	Frailty diff. for in-hospital mortality (solid organ transplant recipients)
**Jung et al. **[52]	Austria, Belgium, Denmark, Egypt, England, France, Germany, Greece, India, Iran, Iraq, Ireland, Israel, Italy, Libya, Mexico, Morocco, Netherland, Norway, Poland, Portugal, Saudi Arabia, Spain, Sudan, Switzerland, USA	19.03.2020–26.05.2020	P/C	CFS	P+	> 70y	PCR	1346	540	75	72–78	965 (71.7)	30-day mortality
**Knights **[53]	UK	01.03.2020–31.03.2020	R/C	CFS	R	n/r	PCR	108	34	68.7	1.5	63 (58)	Frailty diff. for in-hospital mortality
Koduri et al. [54]	UK	20.02.2020–07.05.2020	R/C	CFS	R	> 18y	PCR or Clin + CT	500	193	69,39	17,2	300 (60)	Frailty diff. for in-hospital mort
**Kundi **[55]	Turkey	11.03.2020–22.06.2020	R/C	HFRS	R	> 65	PCR	18,234	3315	74.1	7.4	8498 (46.6)	in-hospital mortality; Frailty diff. for in-hospital mortality; LOH
Kurtz et al. [56]	Brazil	27.02.2020–28.10.2020	P/C	mFI	P	> 18y	PCR	13,301	1785	54	41–69	7752 (58)	30-day mortality
**Lozano-Montoya et al.** [57]	Spain	03.2020–05.2020	R/C	CFS	R	> 75y	PCR or Clin + CT	300	111	86.3	6.6	112 (37.3)	In-hospital mortality
Maguire et al. [58]	UK	18.05.2020–06.07.2020	R/C	CFS	n/r	> 16y	PCR	261	58			119 (46)	30-day mortality
**Maki et al.** [59]	Japan	02.2020–05.2020	R/C	MPI	R	> 65y	PCR	18	4	82.89	10.2	7 (38.9)	In-hospital mortality
**Marengoni **[60]	Italy	08.03.2020–17.04.2020	R/C	CFS	R	n/r	PCR or CT	165	42	69.3	14.5	100 (60.6)	In-hospital mortality, ICU admission
McWilliams [61]	UK	03.2020–04.2020	P/C	CFS	P	> 18	n/a	177	67	n/a	n/a	127 (71.8)	In-hospital mortality, ICU mortality, ICU rehabilitation (only ICU patients)
**Mendes **[62]	Switzerland	13.03.2020–14.04.2020	R/C	CFS	R	> 65	PCR or Clin and Rad	235	76	86.3	6.5	102 (43.4)	In-hospital mortality, frailty diff. for in-hospital mortality
**Moledina **[63]	UK	23.03.2020–07.04.2020	R/C	CFS	R	n/r	PCR	229	75	73	56–81	144 (63)	Frailty diff. for 30-day mortality
**Moloney** [64]	Ireland	17.02.2020–24.04.2020	R/C	CFS	R	> 70	PCR	69	16	79	75–85	40	In-hospital mortality; symptoms, COVID-19 severity, radiological findings, ventilation
**Navaratnam et al.** [65]	UK	01.03.2020–31.05.2020	R/C	HFRS	R	> 18y	PCR	91,541	28,200			50,668 (55.4)	In-hospital mortality
**Noble et al.** [66]	UK	03.2020–06.2020	R/C	CFS	n/r	> 18y	PCR or Clin	164	68	62.1		110 (61.1)	in-hospital mortality
**Osuafor **[67]	UK	01.03.2020–15.05.2020	R/C	CFS	R	> 65	PCR or Clin	214	74	80.3	8.3	120 (56.1)	In-hospital mortality, ICU admission, LOH, readmission; delirium, mobility at discharge, prolonged LOH, death within 14 days of discharge
**Owen** [68]	UK	29.02.2020–16.04.2020	R/C	CFS	R	> 65	PCR	206	92	78.8	8.3	n/a	30-day mortality, ICU admission, ICU mortality
**Piers **[69]	Belgium	03.2020–04.2020	R/C	CFS	R	> 80	n/a	711	246	n/a	n/a	n/a	In-hospital mortality, ICU admission
**Pilotto et al.** [70]	Italy	31.01.2020–31.12.2020	P/C	MPI	P	> 65y	PCR	227	43	80.5		93 (41)	In-hospital mortality
**Ponsford et al.** [71]	UK	01.03.2020–01.07.2020	R/C	CFS	P	> 18y	PCR	2508	885	74	62.5–85.5	1363 (54.3)	In-hospital mortality
**Ramos-Rincon et al.** [72]	Spain	03.03.2020–02.05.2020	R/C	CFS, HFRS	R	> 18y	PCR	290	48				In-hospital mortality; ICU admission; LOH
**Sablerolles et al.** [73]	Austria, Belgium, Denmark, France, Germany, Italy, Netherlands, Portugal, Spain, Switzerland, UK	30.03.2020–15.07.2020	R/C	CFS	P	> 18y	PCR or Clin + CT	2434	456	67	55–77	1480 (61)	In-hospital mortality;ICU admission
Steinmeyer [74]	France	13.03.2020–04.05.2020	R/C	FIND	R	n/r	PCR or Clin and CT	94	17	85.5	7.5	42 (44.6)	In-hospital mortality
**Straw** [75]	UK	05.03.2020–07.05.2020	R/C	CFS	R	> 18	PCR	485	159	71.2	16.9	259 (45.8)	Frailty diff. for in-hospital mortality
**Tehrani** [76]	Sweden	05.03.2020–28.04.2020	R/C	CFS	R	n/r	PCR	255	70	66	17	150 (59)	In-hospital mortality, ventilation
**Thiam et al.** [77]	Malaysia	25.02.2020–27.05.2020	R/C	CFS	R	> 60y	Clin	26	6	76.2	8.2	11 (42.3)	In-hospital mortality
van Steenkiste et al. [78]	Netherland	09.03.2020–01.05.2020	R/C	CFS	R	> 18y	n/r	32	24	79	74.5–83	22 (69)	Frailty diff. for in-hospital mort
**Verholt et al.** [79]	Denmark	01.03.2020–31.05.2020	R/C	MPI	R	> 75y	Clin	100	37	82	77–84	44 (44)	In-hospital mortality; 30-day and 90-day mortality
**Welch et al. **[80]	Egypt, Spain, UK, Greece Ireland, Iraq, Italy, Libya, Saudi Arabia, Sudan, Turkey, USA	n/r	P/C + R/C	CFS	R/P	> 18y	PCR or Clin	5711	1596	74	58–83	3149 (55.1)	In-hospital mortality
**Wolfisberg et al.** [81]	Switzerland	26.02.2020–30.04.2020 and 01.10.2020–31.12.2020	R/C	CFS	R/P	> 18y	PCR or Clin + rAT	486	92	65.9	14.7	317 (65.2)	In-hospital mortality; frailty diff. for in-hospital mort.; ICU admission

Highlighted studies are included in the quantitative analyses. Age is reported using mean ± SD, or median (IQ 1–3) except: Davis P.R. where data was reported as mean (range). The method of frailty assessment was indicated by P for prospective and R in case of retrospective assessment. In case of prospective frailty assessment, if information about the training of the assessor was disclosed, it is marked by ‘+’

*n/a* not available, *n/r* no restriction, *P/C* prospective cohort, *R/C* retrospective cohort, *CFS* Clinical Frailty Scale, *HFRS* Hospital Frailty Risk Score, *FiND* Frail Non-Disabled questionnaire, *PCR* polymerase chain reaction, *Clin* diagnosis based on clinical suspicion, *Rad* radiologically suspected diagnosis, *CT* computer tomography based diagnosis, *CXR* chest X-rax, *rAT* rapid antigen test, *SD* standard deviation, *IQ* interquartile, *OR* odds ratio, *ICU* intensive care unit, *LOH* length of hospitalization, *UK* United Kingdom

### Risk of bias

Risk of bias was assessed separately for in-hospital and 30-day mortality, difference in frailty score for in-hospital and 30-day mortality, ICU admission, and LOH. Most studies did not report detailed baseline data for the frailty groups, therefore carried a high risk of bias (Additional file 1: Fig. S1–S6).

### Frailty is associated with an increased chance of mortality

#### Frailty measured with the Clinical Frailty Scale

46 studies reported on CFS as a measure of frailty. The investigated cohort was dominated by patients from the United Kingdom (UK) in 20 included studies. Most studies reported on in-hospital mortality. Since the CFS represents a continuous spectrum, without evidence for definitive cutoff values, most included studies showed arbitrary partitioning of the CFS. Therefore, we sought to perform quantitative analyses with three distinct partitions (CFS 1–3 vs 4–9, CFS 1–4 vs 5–9, CFS 1–5 vs 6–9). In each of these divisions we performed three different subgroup analyses. Given that a substantial number of patients were from the UK, we divided studies from the UK versus studies outside the UK. Furthermore, the CFS was only validated for patients older than 65 years; we grouped studies accordingly, whether they included patients below 65 years or not. It is important to note that although there was no age restriction at inclusion in some studies, most patients were older than 65 years of age. Beyond that, to further evaluate statistical homogeneity we also undertook a subgroup analysis of assessed mortality (in-hospital vs 30 days). All analyses indicated significant results.

**Fig. 2:**
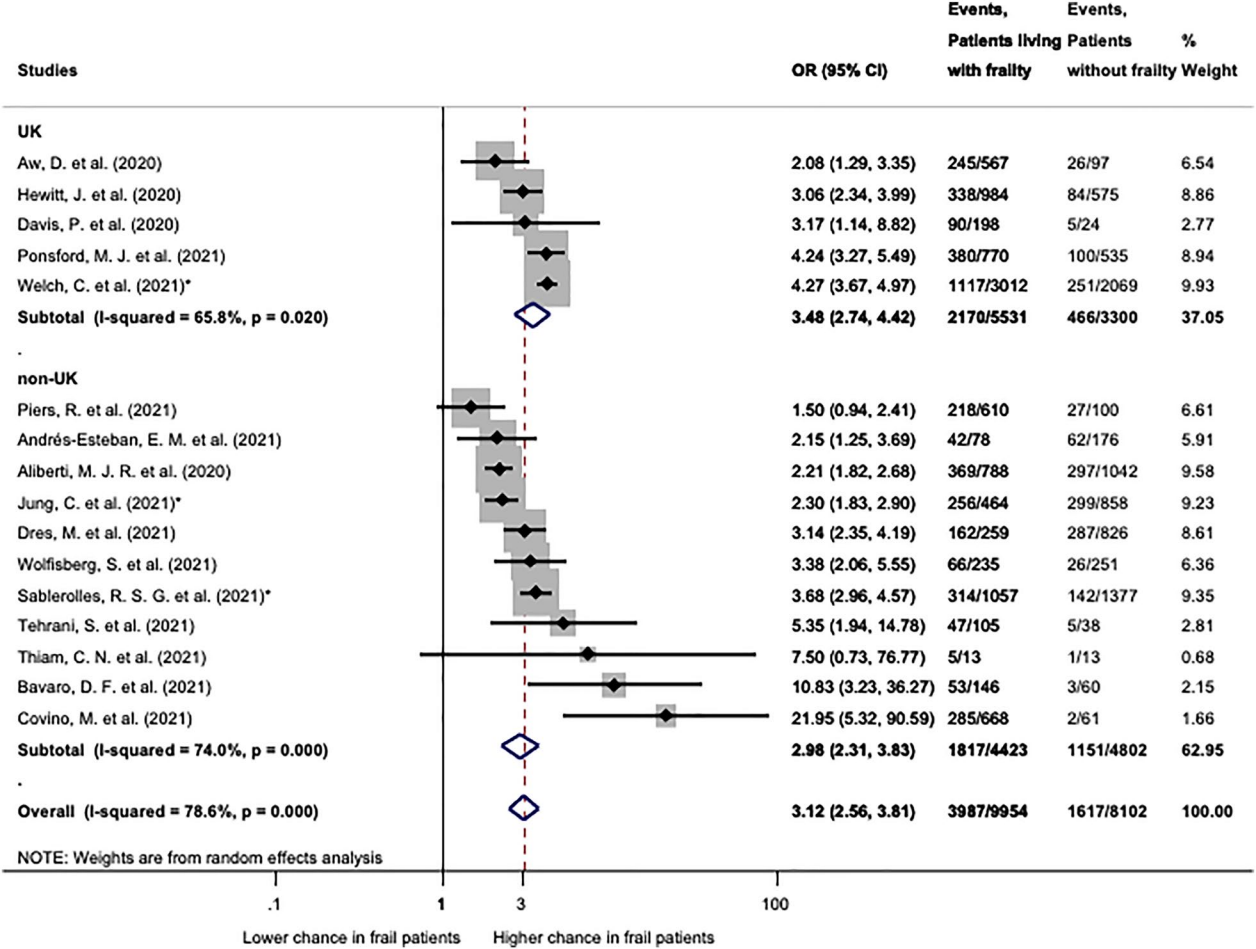
Mortality in patients with frailty (CFS 4–9) compared to not frail (CFS 1–3). Patients living with frailty (CFS 4–9) had significantly higher chance of mortality in both groups (UK and non-UK) and overall. Note that heterogeneity was significant in all cases. OR: odds ratio; CI: confidence interval. *p* < 0.1 was considered significant. *Indicates multicentric studies including patients from both groups, but the majority of patients affiliate to the correspondent subgroup

Quantitative synthesis was performed for studies presenting data on mortality in patients living with frailty (CFS 4–9) compared to patients living without frailty (CFS 1–3) (Fig. 2). Sixteen studies were included in this analysis. Patients with CFS 4–9 had a significantly higher chance of mortality both in the UK subgroup (OR: 3.48; CI 2.74–4.42) and in the non-UK subgroup (OR: 2.98; CI 2.31–3.83) as compared to fit patients (CFS 1–3). In overall patients with CFS 4–9 had significantly, 3.12 times, higher odds for mortality (CI 2.56–3.81) than patients without frailty.

In the analysis regrouped by age restriction at inclusion (Additional file 1: Fig. S8) studies including solely patients 65 years or older demonstrated significant odds for mortality (OR: 3.09; CI 2.08–4.60), as well as studies without age restriction (OR: 3.27; CI 2.56–3.81) (CFS 4–9 vs 1–3). The regrouped analysis separating studies reporting in-hospital and 30-day mortality (Additional file 1: Fig. S7) showed significant results in both groups: OR: 3.39 (CI 2.70–4.26) and OR: 2.46 (CI 2.07–2.93) for in-hospital and 30-day mortality, respectively. However, assessing 30-day mortality demonstrated increasing statistical homogeneity (*I*^2^ = 31.5%, *p* = 0.223) as compared to in-hospital mortality (*I*^2^ = 72.6%, *p* = 0.000). No influential study was identified by the leave-one-out sensitivity analysis (Additional file 1: Fig. S9).

**Fig. 3:**
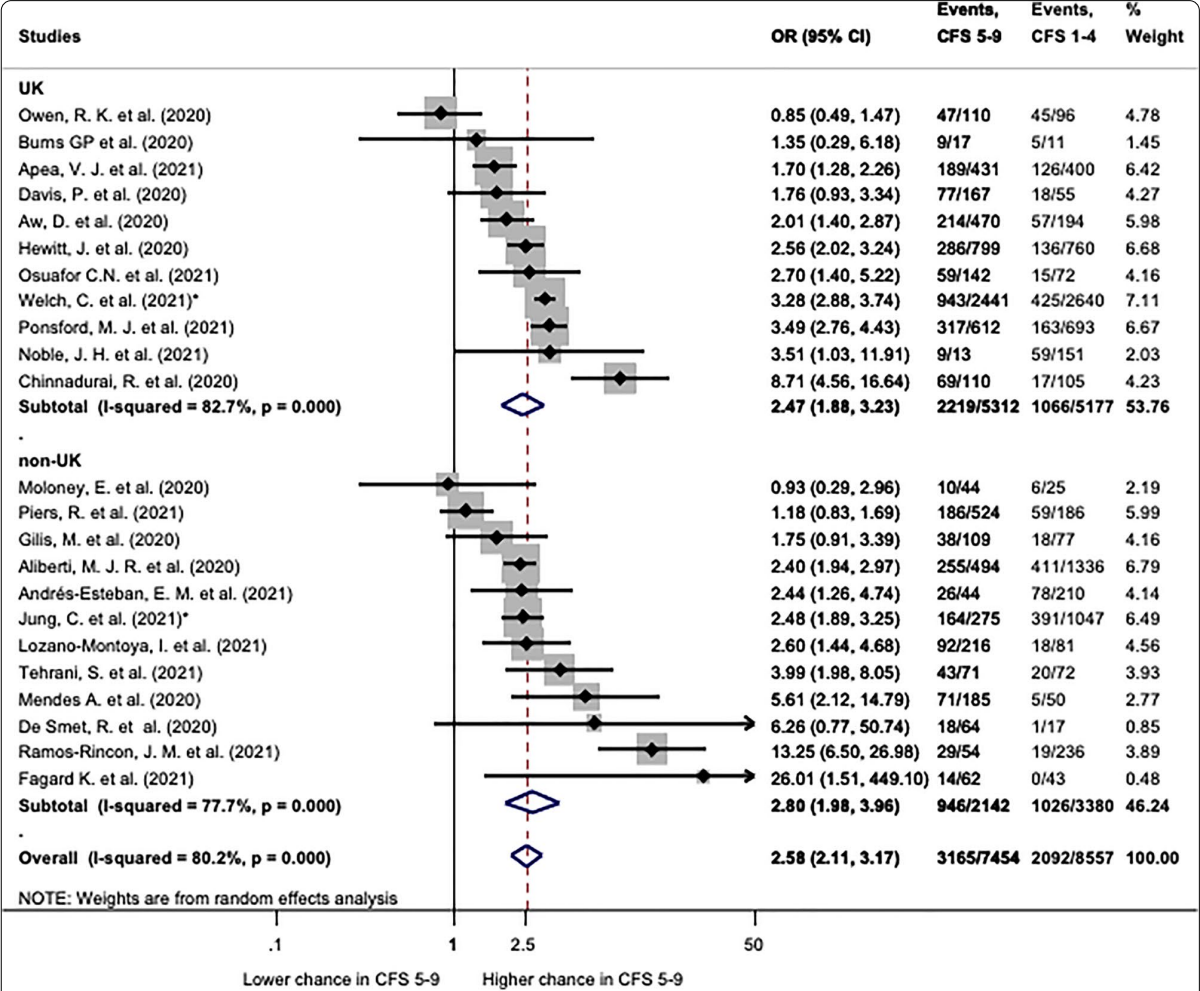
Mortality in patients with CFS 5–9 compared to CFS 1–4. Patients with CFS 5–9 have significantly higher odds of mortality (OR: 2.58; CI 2.11–3.17). Patients from the UK (OR: 2.47; CI 1.88–3.23) and non-UK (OR: 2.80; CI 1.98–3.96) had significantly higher odds as well. Note that heterogeneity was significant in all cases. OR: odds ratio; CI: confidence interval. *p* < 0.1 was considered significant. *Indicate multicentric studies including patients from both groups, but the majority of patients affiliate to the correspondent subgroup

In order to get a more thorough picture, studies comparing CFS 1–4 with CFS 5–9 were also quantitatively analysed (Fig. 3). This is in line with the original classification of the CFS, where a score greater than 4 indicated frailty [2]. Twenty-three studies presented sufficient data for this analysis. Frailty represented as CFS 5–9 demonstrated still significantly higher odds ratio for mortality: 2.58 (CI 2.11–3.17) as compared to CFS 1–4 (Fig. 3). Patients from the UK and from other countries had comparable odds ratios 2.47 (CI 1.88–3.23) and 2.58 (CI 2.11–3.17), respectively (Fig. 3). Analysing subgroups with and without age restriction (Additional file 1: Fig. S12), and 30-day and in-hospital mortality (Additional file 1: Fig. S11) could not achieve homogenization of results. All assessed subgroups showed significant heterogeneity, but an influential study could not be identified by the leave-one-out sensitivity analysis (Additional file 1: Fig. S13).

Patients with CFS 1–5 and CFS 6–9 were also compared (Additional file 1: Figs. S15–S17). The overall odds ratio for mortality was 2.55 (CI 2.13–3.07). Again, all subgroups demonstrated significant results, but heterogeneity was significant in all cases, although no influential study was identified by the leave-one-out sensitivity analysis (Additional file 1: Fig. S18).

Similarly to our results, multiple logistic regression adjusted for age, sex, respiratory rate, FiO_2_, consolidation, and urea resulted in an OR of 2.55 (CI 1.74–3.74) for 30-day mortality and OR: 2.60 (CI 1.34–5.06) for 72-h mortality by Bradley et al. for patients with CFS ≥ 5 [82].

Maguire et al. reported on a retrospective cohort of 261 patients. Unfortunately the presented data was contradictory, thus could not be included in the quantitative synthesis.

Nineteen studies reported the mean or median frailty in survivors and non-survivors, of which 12 were included in quantitative synthesis (Additional file 1: Fig. S21). Non-survivors generally scored significantly higher using the CFS than survivors (overall WMD: 1.21; CI 0.83–1.59). Differences were significant for in-hospital and 30-day mortality separately. Regrouping by country also yielded significant results in both subgroups (Additional file 1: Fig. S20). No influential study was identified by the leave-one-out sensitivity analysis (Additional file 1: Fig. S22).

Similarly to the results of the quantitative synthesis, Brill et al. reported, that the median CFS was 4 in discharged patients versus 5 in patients who died (*p* = 0.014) [37].

Cecchini et al. reported on a hospital cohort of 122 geriatric patients [39]. Median CFS was significantly higher in non-survivors than survivors (7 vs 6, respectively; *p* = 0.001). IQR was not appropriately stated, thus could not be included in the analysis. Cuvelier et al. included 20 severe COVID-19 patients admitted to a geriatric intermediate care unit, who were not eligible for any higher level treatment [42]. Non-survivors had higher median CFS (6.0, IQR: 5.5–6.5) compared to survivors (4.5, IQR: 3.5–6.0). In both groups two patients had missing CFS data. Fallon et al. reported on a hospital cohort of 86 elderly patients [47]. Non-survivors had a mean CFS of 5.2 compared to survivors’ 4.1 (SD was not published). Hoek et al. provided data on solid organ transplant recipients. The mean CFS was 5.8 points for patients who died, while 1.92 points for survivors (SD was not disclosed) [51]. Koduri et al. reported on a single center cohort of 500 patients [54]. Non-survivors had significantly higher median CFS score (5, min: 1, max: 9) than survivors (3, min: 1, max: 9), (*p* < 0.001).

McWilliams et al. only included COVID-19 patients admitted to the ICU, therefore could not be pooled. ICU mortality and hospital discharge destination were detailed by CFS score categories [61]. 67 patients died in the ICU, who’s CFS score was significantly higher than ICU survivors’ (*p* < 0.001). Only one patient died in the hospital after ICU discharge, who’s CFS score is not detailed.

Van Steenkiste et al. included 32 severe patients, who were not deemed eligible for invasive mechanical ventilation and received high-flow nasal oxygen therapy as a rescue [78]. There was no difference in median CFS score between survivors and non-survivors (4, IQR: 4–6 vs 4, IQR: 4–6, *p* = 0.44).

#### Frailty measured by the Hospital Frailty Risk Score

**Fig. 4:**
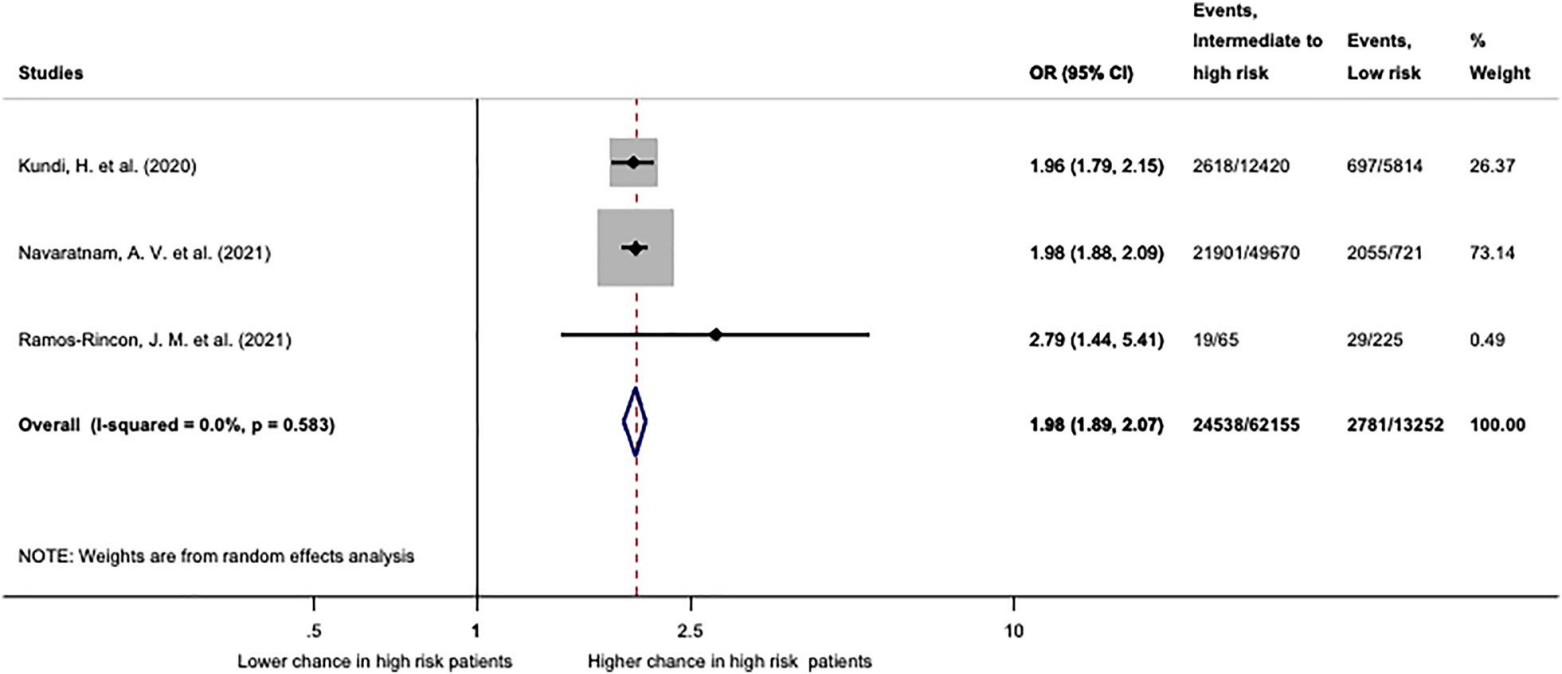
Mortality assessed by the Hospital Frailty Risk Score. Patients with intermediate and high risk (HFRS ≥ 5) have significantly higher odds of mortality (OR: 1.98; CI 1.89–2.07) compared to patients with low risk of frailty (HFRS < 5). This analysis was statistically homogeneous (*I*^2^ = 0.0%, *p* = 0.583). OR: odds ratio; CI: confidence interval. *p* < 0.1 was considered significant

We performed a quantitative synthesis of three studies reporting mortality in patients living with frailty using Hospital Frailty Risk Score (Fig. 4) Two of these studies analysed nationwide recorded electronic databases (Navaratnam et al. from England and Kundi et al. from Turkey) including over 75,000 patients. The third study was a hospital cohort study from Spain (Ramos-Rincon et al.). Compared to the low-risk group (HFRS < 5) patients with intermediate and high risk of frailty had significantly higher chance for mortality (OR: 1.98; CI 1.89–2.07). Results were statistically homogenous (*I*^2^ = 0.0%; *p* = 0.583).

Apea et al. reported HFRS in a cohort from five acute hospitals in London. Since there is high potential for an overlapping patient population with Navaratnam et al., Apea et al. has been excluded from this analysis. Based on their data calculated odds ratio for mortality was 5.21 (CI 4.03–6.74) in the intermediate and high-risk group (HFRS ≥ 5) compared to the low-risk group (HFRS < 5).

#### Frailty measured by the Multidimensional Prognostic Index

Quantitative synthesis of three studies reporting on mortality associated with frailty measured by the MPI was undertaken (Additional file 1: Fig. S24). Contradictorily, in the study by Maki et al. mortality in patients with frailty (MPI 2 and 3) was 16.7% as compared to 33.3% in patients without frailty (MPI 1) [59]. This could be due to the very low sample size (*n* = 18). Overall odds ratio for mortality in MPI 2 + 3 compared to MPI 1 was 4.31 (CI 0.91–20.49) but did not reach statistical significance. Although all studies reported in-hospital mortality, there was significant, substantial heterogeneity (*I*^2^ = 68.8%, *p* = 0.041).

Verholt et al. also reported significantly higher chance of 30-day and 90-day mortality in patients living with frailty (MPI 2 + 3) in contrast to patients with MPI 1 [79].

#### Frailty measured by miscellaneous tools

Two studies presented data on mortality in association with a modified frailty index (mFI) [48, 56]. Fumagalli et al. included 221 patients aged 75 or older from two centres [48]. 44.3% of deceased patients were frail in contrast to 29% in survivors. The absence of frailty was significantly associated with survival (adjusted HR 0.6; CI 0.39–0.94; *p* = 0.024). Kurtz et al. presented data on 13,301 patients. Frailty indicated by mFI was associated with worse 30-day and 60-day survival. (MFI > 2 60-day mortality HR: 1.38; CI 1.15–1.64; *p* < 0.001) [56].

Steinmeyer et al. reported on a geriatric cohort of patients, where frailty was assessed with the Frail Non-Disabled Survey (FIND). According to their analysis frailty was not correlated with mortality [74].

### ICU admission

Ten studies reported on the association of frailty indicated by the CFS and ICU admission. We conducted two analyses with different partition of CFS (1–3 vs 4–9 and 1–4 vs 5–9). Due to the arbitrary grouping of the CFS by different authors 7 and 6 studies could be included in the analyses of CFS 1–3 vs 4–9 and CFS 1–4 vs 5–9, respectively.

**Fig. 5:**
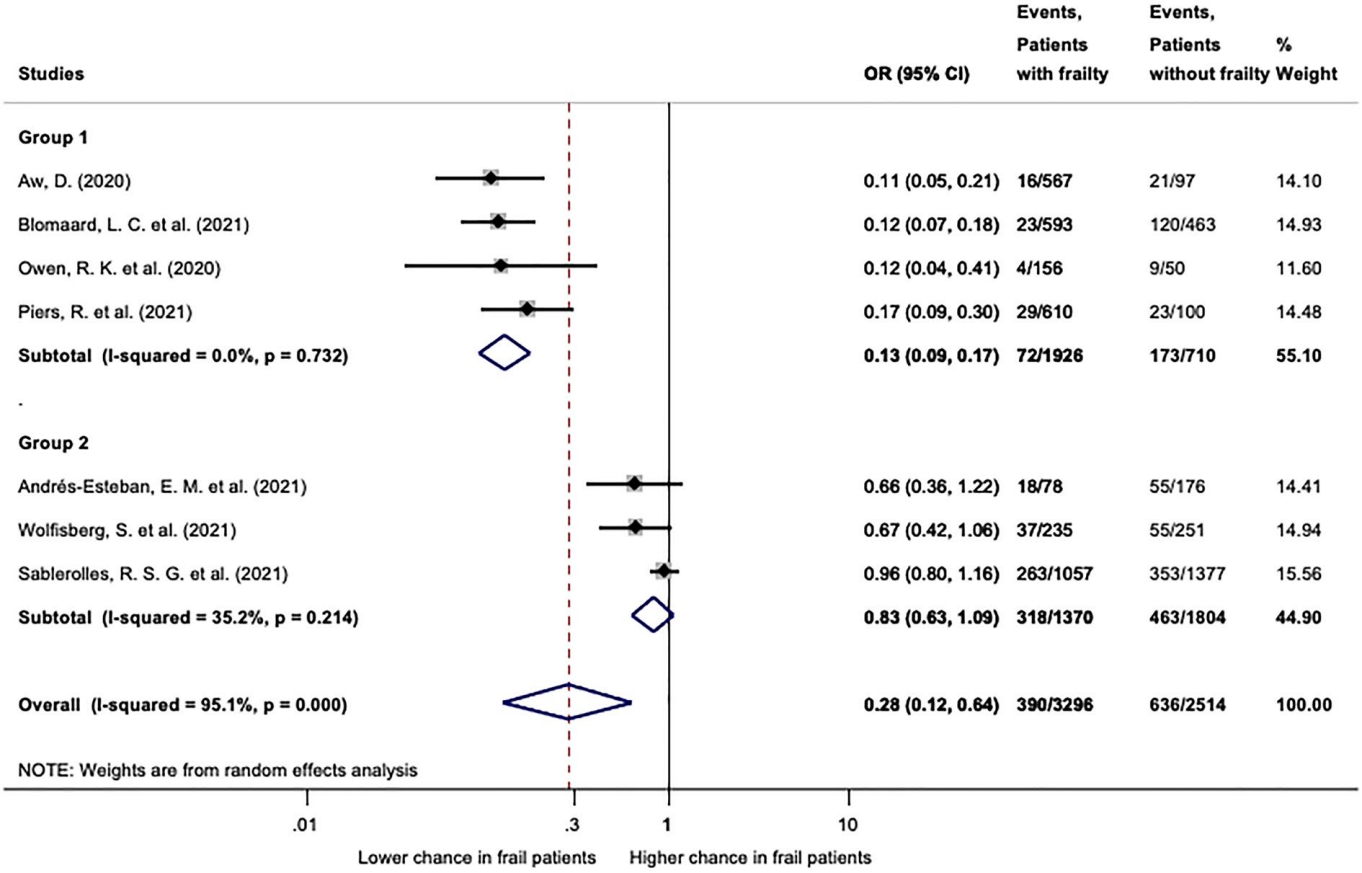
ICU admission in patients with frailty indicated by CFS 1–3 vs 4–9. Patients living with frailty (CFS 4–9) have significantly lower odds to be admitted to the ICU (overall OR: 0.28; CI 0.12–0.64). In group 1 chance for ICU admission was significantly lower in patients with frailty (OR: 0.13; CI 0.09–0.17), however in group 2 to there was no significant difference (OR: 0.83; CI 0.63–1.09). For further explanation please see text. Please note, that in contrast to the significant overall heterogeneity, both subgroups were statistically homogeneous. OR: odds ratio; CI: confidence interval. *p* < 0.1 was considered significant

The analysis of CFS 1–3 vs 4–9 resulted in an overall odds ratio of 0.28 (CI 0.12–0.64), although displaying in considerable statistical heterogeneity (*I*^2^ = 95.1%, *p* = 0.000). In order to clarify possible reasons behind this, we divided the pool in two distinct subgroups. This resulted in statistical homogeneity on both groups (*I*^2^ = 0.0%, *p* = 0.732 and *I*^2^ = 35.2%, *p* = 0.214) (Fig. 5). One possible explanation might be that studies included in the first group originated from countries, where CFS was included in guidelines for advanced care planning (Belgium, the Netherlands, the UK). Here frailty resulted in significantly lower chance of ICU admission (OR: 0.13; CI 0.09–0.17). In contrast, frailty based advanced care planning was not applied in majority of centres included in the second group. It is to note, that Sablerolles et al. is a European multicentric retrospective cohort study in which centres from Belgium, the Netherlands, France and the UK recruited patients as well, but more than 50% of the included patients originated from other European countries. In this group frailty did not significantly reduce chance for ICU admission (OR: 0.83; CI 0.63–1.09) (Fig. 5). The leave-one-out sensitivity analysis did not identify any influential study (Additional file 1: Fig. S25).

In another quantitative analysis of studies, we examined association of ICU admission in a patient group with further advanced frailty (CFS 5–9) compared to robust and very mildly frail patients (CFS 1–4). In the quantitative synthesis 6 studies could be included (Additional file 1: Fig. S27). In overall, advanced frailty (CFS 5–9) resulted in significantly lower chance of ICU admission (OR: 0.09; CI 0.04–0.22). Although a subgroup analysis was not applicable, an analogical tendency can be observed as in Fig. 5, resulting in significant overall heterogeneity (*I*^2^ = 64.9%, *p* = 0.014). The leave-one-out sensitivity analysis did not identify any influential study (Additional file 1: Fig. S28).

### Length of stay

The average length of stay was reported in seven studies. Five studies reported on CFS, one study on HFRS and one on both. Arbitrary categorization and different statistical methods of data presentation made quantitative analyses unfeasible. A brief summary of results can be found in Additional file 1: P. 34) The observed outcomes show substantial heterogeneity and no meaningful, generalizable conclusion can be drawn.

### Publication bias

Eggers’s test was only conducted where at least 10 studies were included in the analysis. Visual examination of funnel plots and Eggers’ tests did not show small-study effect for any examined outcomes, but one. (Additional file 1: Figs. S10, S14, S19, S23, S24, S26, S29). On the funnel plot of ICU admission CFS 1–3 vs 4–9 (Additional file 1: Fig. S10) strong asymmetry can be observed, which may be due to publication bias. HFRS and MPI could not be examined due to the low number of studies included in the analyses.

## Discussion

In this systematic review and meta-analysis on the relationship between frailty and mortality, ICU admission, and LOH in COVID-19 patients, with the inclusion of 54 studies and 152,628 subjects, we found that frailty was associated with significantly elevated odds for mortality and frail patients were less likely to be admitted to the ICU.

Despite advances in critical care management, mortality of severe respiratory failure especially with COVID-19 remains high [83–85]. Advanced organ support—the cornerstone of intensive care—may interfere with human dignity. The relatively high mortality and the required work intensity means a burden for the patient, relatives, and staff alike and is—last but not least—costly [86, 87]. Therefore, prolonged, advanced organ support might be regarded as medically futile in those cases, whose chances are extremely limited for survival [88, 89], hence predictors of survival have been extensively researched. Due to the unprecedented load on ICUs during the pandemic of COVID-19, implementing a reliable tool to identify those who could not benefit from intensive care would be of utmost help for clinicians, patients, and relatives alike.

It is well known that age on its own can be misleading in outcome prediction [90]. A potential alternative is the assessment of frailty, a concept that has already been supported during the COVID-19 pandemic by some studies and recent meta-analyses [12–19]. However, multiple methodological flaws were detected in previous meta-analyses, such as pooling of odds, risk and hazard ratios, as well as pooling of different frailty tools together [13, 19]. In terms of CFS we disagree with the calculation of dose response, while single CFS increments cannot be compared [14]. Furthermore, Kastora et al. demonstrated a variable increase in mortality between single CFS increments [17]. In most of those studies reporting on CFS, categorization was arbitrary and the authors did not report outcomes for each CFS score except five included studies [43, 50, 52, 69, 71]. In contrast to recent meta-analyses, we sought to analyse the most meaningful arrangements of CFS groups dividing patients into a fit to minimal degree of frailty group compared to patients with more advanced level of frailty. Consequently, we only included studies into each analysis, which reported data on the respective grouping.

It is important to note, that frailty assessment-based decision-making has not been implemented worldwide. According to our literature search (up to September 2021), several countries (UK, the Netherlands, Belgium, and France), with well resourced, high-quality health care advised the use of CFS in decision-making in their COVID-19 guidelines; in contrast to Central and Eastern Europe [91–93].

Limitation of treatment, i.e.: denying advanced levels of care to patients based on the level of frailty might increase mortality, in a way of a self-fulfilling prophecy. As a remarkable portion of the studies included in our meta-analysis originated from countries where frailty-based ceiling of care decision-making protocols are already implemented, this fact on its own can influence the observed mortality and therefore our results as well. However, there is an increasing body of evidence from other countries where frailty-based treatment limitations are not included in daily routine patient management. Kundi et al. reports in a Turkish nation-wide assessment that high-risk frail patients (Hospital Frailty Risk Score > 15) had higher odds for all-cause mortality (adjusted OR: 2.084; CI 1.799–2.413) but also a higher chance for ICU admission (adjusted OR: 2.221; CI 1.951–2.527), as well as receiving invasive mechanical ventilation (adjusted OR: 1.769; CI 1.531–2.046) [55]. In a large European multicentric retrospective study Sablerolles and colleagues provided evidence that compared to fit patients (CFS 1–3), patients with frailty (CFS 6–9) had a significantly higher chance of ICU admission (adjusted OR: 1.54; CI 1.21–1.97), and in addition those admitted to the ICU were significantly more likely to die (OR: 1.81; CI 1.14–2.87) [73]. These findings align with our results, suggesting that although there might be some effect of limiting higher level of care, the observed high mortality rate in frail COVID-19 patients cannot merely be explained by that. Furthermore, our results also suggest that measuring frailty could potentially help in the selection process of those patients who could not benefit from intensive care.

As the clinically more aggressive variants are spreading across the world, including Eastern and Central Europe—the home region of the authors—the effective allocation of resources would be of utmost importance. These countries were more-or-less spared during the first wave and also in the second wave when mortality rates were higher in Western and Northern European countries [94–96]. However, the third wave proved devastating in this region of Europe from both the ICU-burden and the survival perspectives [97].

Although ethical concerns were raised against frailty-based decision-making, this method potentially provides a professional and transparent scaffold for health care providers [98, 99]. It has also been shown that the decision to withhold or withdraw life-sustaining treatment from patients older than 80 years in the ICU correlates with income and religious influence. In countries with lower income and higher religiosity, high-intensity critical care treatments are less frequently withdrawn, and the decision does not depend on age and ICU bed availability [100]. In a recent multicentre, multinational prospective observational study on 1346 older adult (> 70) ICU COVID-19 patients, frailty provided relevant prognostic information in addition to age and comorbidities [52]. Furthermore, an indirect comparison by Kow et al. has indicated that frail individuals may be overrepresented among the COVID-19 patient population and given a rather strong hint that the presence of frailty may lead to a higher risk of acquisition of COVID-19 [101]. In addition to frailty, severity of the acute illness also has a major role in clinical outcomes, especially in the elderly, more frail population. It is also important to note that patient care pathways could also have an impact on the final outcome, however, detailed discussion of this issue is beyond the scope of the current manuscript.

Finally, it should be mentioned that frailty must never be applied as a stand-alone cutoff value in patient management. However, it should be part of a patient based, individualized decision making. Therefore it would be desirable that based on the available scientific evidence, health authorities encouraged and supported the implementation of frailty-based risk assessment into national guidelines.

## Strengths and limitations

To our knowledge, this is the most detailed and comprehensive, up to date evaluation of frailty in COVID-19, separately analysing 30-day and in-hospital mortality, studies from and outside of the UK and reporting on different age groups, as well as including five different frailty assessment scores. We also assessed the relationship between frailty and ICU admission. In contrast to other meta-analyses, we only pooled together studies using the same frailty score, same frailty range, and similar statistical tools. Another methodological strength is that leave-one-out sensitivity analysis was also performed to identify influential studies. The majority of the included studies were retrospective and carried high risk of bias, therefore could introduce bias in our analysis. Nevertheless, one of the most important limitations of our study is the considerable heterogeneity that was a common feature in many of the analyses. The explanation could lie in standard medical practice, age distribution, and nurse-to-patient ratio, which can differ between countries and hospitals. Another limitation is that we did not have access to individual patient data. Furthermore, studies implementing frailty assessment in COVID-19 were not published from Central and Eastern European countries; therefore, they were not represented in the quantitative analyses.

## Conclusion

To the best of our knowledge, this is the largest, most recent and comprehensive meta-analysis of studies in this topic today in COVID-19 patients. Our results show that frailty as determined by CFS is strongly associated with in-hospital and 30-day mortality and may also play an important role in determining eligibility for ICU admission in patients suffering from COVID-19. These findings have some implications for research: further evaluation of the effects of frailty-based patient management on ICU admission, ICU mortality as well as long term outcomes should also be investigated in the future within the scope of high-quality, low risk of bias studies. Regarding implications for practice, we believe that frailty-based patient management should be included in international COVID-19 treatment guidelines.

## Supplementary Information

**Additional file 1.** Risk of bias assessment protocol. **Figure S1.** Risk of bias assessment for in-hospital mortality. **Figure S2.** Risk of bias assessment for 30-day mortality. **Figure S3.** Risk of bias assessment for average frailty comparing deceased and discharged COVID-19 patients. **Figure S4.** Risk of bias assessment for average frailty comparing COVID-19 patients who survived for 30-days vs who did not survive. **Figure S5.** Risk of bias assessment for ICU admission. **Figure S6.** Risk of bias assessment for length of hospital stay. **Figure S7.** 30-day and in-hospital mortality in patients with frailty indicated by CFS (1–3 vs 4–9). **Figure S8.** Mortality in frail patients indicated by CFS (1–3 vs 4–9), with studies grouped by age restriction. **Figure S9.** Leave-one-out sensitivity analysis for studies reporting mortality in patients with CFS 1–3 vs 4–9. **Figure S10.** Funnel plot for mortality in patients with CFS 1–3 vs 4–9. **Figure S11.** 30-day and in-hospital mortality in patients with CFS 1–4 vs 5–9. **Figure S12.** Mortality in patients with CFS 1–4 vs 5–9, with studies grouped by age restriction. **Figure S13.** Leave-one-out sensitivity analysis for studies reporting mortality in patients with CFS 1–4 vs 5–9. **Figure S14.** Funnel plot for mortality in patients with CFS 1–4 vs 5–9. **Figure S15.** Mortality comparing CFS 1–5 and CFS 6–9 groups, with studies grouped by country. **Figure S16.** 30-day and in-hospital mortality in patients with CFS 1–5 vs 6–9. **Figure S17.** Mortality comparing CFS 1–5 and CFS 6–9 groups, with studies grouped by age restriction. **Figure S18.** Leave-one-out sensitivity analysis for studies reporting mortality in patients with CFS 1–5 vs CFS 6–9. **Figure S19.** Funnel plot for mortality in patients with CFS 1–5 vs 6–9. **Figure S20.** Forest plot for weighted mean difference of CFS score for mortality with studies grouped by country. **Figure S21.** Forest plot for weighted mean difference of CFS score for mortality with studies grouped by follow-up. **Figure S22.** Leave-one-out sensitivity analysis for studies reporting average frailty indicated by CFS in survivors and non-survivors. **Figure S23.** Funnel plot for frailty difference in survivors vs non-survivors. **Figure S24.** Forest plot for mortality in patients with MPI 1 vs 2 and 3. **Figure S25.** Leave-one-out sensitivity analysis for studies reporting ICU admission in patients with CFS 1–3 vs 4–9. **Figure S26.** Funnel plot for ICU admission in patients with CFS 1–3 vs 4–9. **Figure S27.** Forest plot for ICU admission in patients with CFS 1–4 vs 5–9. **Figure S28.** Leave-one-out sensitivity analysis for studies reporting ICU admission in patients with CFS 1–4 vs 5–9. **Figure S29.** Funnel plot for ICU admission in patients with CFS 1–4 vs 5–9. Length of hospital stay: Summary of reported results.

**Additional file 2.** PRISMA 2020 checklist.

## Data Availability

Original data is available from the corresponding author on reasonable request.

## Abbreviations

ICU: Intensive care unit
LOH: Length of hospitalization
CENTRAL: The Cochrane Central Register of Controlled Trials
QUIPS: Quality in Prognosis Studies tool
OR: Odds ratio
WMD: Weighted mean difference
CI: Confidence interval
CFS: Clinical Frailty Scale
HFRS: Hospital Frailty Risk Score
MPI: Multidimensional Prognostic Index
ICD: International Classification of Diseases
PRISMA: Preferred Reporting Items for Systematic Reviews and Meta-Analyses
PECO: Patient, Exposure, Control, Outcome
SD: Standard deviation
FIND: Frail Non-Disabled questionnaire
IQ: Interquartile
UK: United Kingdom
n/a: Not available
n/r: No restriction
P/C: Prospective cohort
R/C: Retrospective cohort
PCR: Polymerase chain reaction
Clin: Clinical
Rad: Radiological
CT: Computer tomography
CXR: Chest X-ray
rAT: Rapid antigen testing
FiO_2_: Fraction of inspired oxygen
