# Supplementary material for: Clinical Frailty Scale (CFS) indicated frailty is associated with increased in-hospital and 30-day mortality in COVID-19 patients: a systematic review and meta-analysis
Source: Ann Intensive Care. 2022 Feb 20;12:17. doi: 10.1186/s13613-021-00977-4 (PMC8858439; doi:10.1186/s13613-021-00977-4)
Supplement: Supplementary file 1 — Additional file 1. Risk of bias assessment protocol. Figure S1. Risk of bias assessment for in-hospital mortality. Figure S2. Risk of bias assessment for 30-day mortality. Figure S3. Risk of bias assessment for average frailty comparing deceased and discharged COVID-19 patients. Figure S4. Risk of bias assessment for average frailty comparing COVID-19 patients who survived for 30-days vs who did not survive. Figure S5. Risk of bias assessment for ICU admission. Figure S6. Risk of bias assessment for length of hospital stay. Figure S7. 30-day and in-hospital mortality in patients with frailty indicated by CFS (1–3 vs 4–9). Figure S8. Mortality in frail patients indicated by CFS (1–3 vs 4–9), with studies grouped by age restriction. Figure S9. Leave-one-out sensitivity analysis for studies reporting mortality in patients with CFS 1–3 vs 4–9. Figure S10. Funnel plot for mortality in patients with CFS 1–3 vs 4–9. Figure S11. 30-day and in-hospital mortality in patients with CFS 1–4 vs 5–9. Figure S12. Mortality in patients with CFS 1–4 vs 5–9, with studies grouped by age restriction. Figure S13. Leave-one-out sensitivity analysis for studies reporting mortality in patients with CFS 1–4 vs 5–9. Figure S14. Funnel plot for mortality in patients with CFS 1–4 vs 5–9. Figure S15. Mortality comparing CFS 1–5 and CFS 6–9 groups, with studies grouped by country. Figure S16. 30-day and in-hospital mortality in patients with CFS 1–5 vs 6–9. Figure S17. Mortality comparing CFS 1–5 and CFS 6–9 groups, with studies grouped by age restriction. Figure S18. Leave-one-out sensitivity analysis for studies reporting mortality in patients with CFS 1–5 vs CFS 6–9. Figure S19. Funnel plot for mortality in patients with CFS 1–5 vs 6–9. Figure S20. Forest plot for weighted mean difference of CFS score for mortality with studies grouped by country. Figure S21. Forest plot for weighted mean difference of CFS score for mortality with studies grouped by follow-up. Figure S22. Leave-one-out sensiti [file 13613_2021_977_MOESM1_ESM.pdf]

Clinical Frailty Scale (CFS) indicated frailty is associated with increased in-hospital and 30-day mortality in COVID-19 patients: a systematic review and meta-analysis

Additional File 2

Máté Rottler*[1,4,5], Klementina Ocskay*[1,2], Zoltán Sipos[1], Anikó Görbe[1], Marcell Virág[1,4,5], Péter Hegyi[1,2,3], Tihamér Molnár[6], Bálint Erőss[1,2,3], Tamás Leiner[1,7], Zsolt Molnár[2,3,5,8,9]

[1] Institute for Translational Medicine, Szentágothai Research Centre, Medical School, University of Pécs, Pécs, Hungary

[2] Centre for Translational Medicine, Semmelweis University, Budapest, Hungary

[3] Division of Pancreatic Diseases, Heart and Vascular Center, Semmelweis University, Budapest, Hungary

[4] Department of Anaesthesiology and Intensive Therapy, Szent György University Teaching Hospital of Fejér County, 8000 Székesfehérvár, Hungary

[5] Doctoral School of Clinical Medicine, University of Szeged, 6720 Szeged, Hungary

[6] Department of Anaesthesiology and Intensive Therapy, University of Pécs, 7624 Pécs

[7] Anaesthetic Department, Hinchingbrooke Hospital, North West Anglia NHS Foundation Trust, Huntingdon PE29 6NT, UK

[8] Department of Anaesthesiology and Intensive Therapy, Poznan University of Medical Sciences, 61-701 Poznan, Poland

[9] Department of Anaesthesiology and Intensive Therapy, Semmelweis University, 1082, Budapest, Hungary

* Authors contributed equally

Correspondence to: Zsolt Molnár; zsoltmolna@gmail.com; Tel: +36-30-302-6668

Table of contents

Risk of bias assessment 4
Risk of bias assessment protocol 4
Figure S1 — In-hospital mortality 5
Figure S2 — 30-day mortality 6
Figure S3 — Average frailty comparing deceased and discharged COVID-19 patients 7
Figure S4 — Average frailty comparing COVID-19 patients who survived for 30-days vs who did not survive 8
Figure S5 — ICU admission 9
Figure S6 — Length of hospital stay 10
Mortality in patients with CFS 1–3 vs 4–9 11
Figure S7 – Forest plot for mortality grouped by follow-up 11
Figure S8 – Forest plot grouped by age restriction 12
Figure S9 – Leave-one-out analysis 13
Figure S10 – Funnel plot 14
Mortality in patients with CFS 1–4 vs 5–9 15
Figure S11 — Forest plot for mortality grouped by follow-up 15
Figure S12 — Forest plot grouped by age restriction 16
Figure S13 – Leave-one-out analysis 17
Figure S14 – Funnel plot 18
Mortality in patients with CFS 1–5 vs 6–9 19
Figure S15 – Forest plot, studies grouped by country 19
Figure S16 — Forest plot for mortality grouped by follow-up 20
Figure S17 — Forest plot grouped by age restriction 21
Figure S18 — Leave-one-out analysis 22
Figure S19 – Funnel plot 23
Weighted Mean Difference of CFS 24
Figure S20 – Forest plot, studies grouped by country 24
Figure S21 – Forest plot for mortality grouped by follow-up 25
Figure S22 — Leave-one-out analysis 26
Figure S23 – Funnel plot 27
Mortality in patients with MPI 1 vs 2+3 28
Figure S24 – Forest plot 28
ICU admission in patients with CFS 1–3 vs 4–9 29

Figure S25 – Leave-one-out analysis 29
Figure S26 – Funnel plot 30
ICU admission in patients with CFS 1–4 vs 5–9 31
Figure S27 – Forest plot 31
Figure S28 – Leave-one-out analysis 32
Figure S29 – Funnel plot 33
Length of hospital stay 34
Summary of reported results 34
References 35

Risk of bias assessment

Risk of bias assessment protocol

Following the recommendations of the Cochrane Collaboration, the Quality in Prognosis Studies (QUIPS) tool was used by MR and TL independently [1]. Disagreements were resolved by ZM. In the study participation domain gender, age, ethnicity and comorbidities were taken into account. Study attrition was not judged for retrospective studies. In the prognostic factor measurement domain, the specification of the frailty assessor, information about their training and missing data on frailty were taken into account. Less than 10% missing data was considered low risk, 10–20% some concerns and more than 20% resulted in high risk for the whole domain. Outcome measurement and statistical analysis domains carried low risk in most cases because mortality is a hard outcome and we mostly used raw data. In case of ICU admission, a detailed protocol for ICU admission was needed. In the study confounding domain, studies reporting baseline information for the frailty groups separately were judged low risk if no clinically significant differences were seen, some concerns if some differences were seen and high risk if no data was reported. The overall risk of bias was calculated using the suggestions of Grooten et al. [2].

Figure S1 — In-hospital mortality

A

n.a. Not applicable
\+ Low risk
? Moderate risk
\- High risk

Tool	Study	Study participation	Study attrition	Prognostic factor measurement	Outcome measurement	Study confounding	Statistical analysis reporting	Overall risk of bias	Included in meta-analyses
CFS	Andrés-Esteban, E. M. et al. (2021)	-	n.a.	+	+	+	+	-	Yes
	Covino, M. et al. (2021)	+		+	+	+	+	+	Yes
	Aw, D. et al. (2020)	+	n.a.	+	+	+	+	+	Yes
	Blomaard, L. C. et al. (2021)	+	n.a.	-	+	?	+	-	Yes
	Burns GP et al. (2020)	?	n.a.	+	+	-	+	-	Yes
	Chinnadurai, R. et al. (2020)	+	n.a.	+	?	-	+	-	Yes
	Bavaro, D. F. et al. (2021)	+	n.a.	?	+	+	+	+	Yes
	De Smet, R. et al. (2020)	+	n.a.	+	+	-	+	-	Yes
	Fagard K. et al. (2021)	?	n.a.	+	+	-	+	-	Yes
	Hewitt, J. et al. (2020)	+	+	+	+	-	+	-	Yes
	Lozano-Montoya, I. et al. (2021)	-	-	-	+	-	+	-	Yes
	Marengoni, A. et al. (2020)	+	n.a.	+	+	-	+	-	Yes
	Mendes A. et al. (2020)	+	n.a.	+	+	-	+	-	Yes
	Moloney, E. et al. (2020)	+	n.a.	+	+	+	+	+	Yes
	Noble, J. H. Et al. (2021)	-		-	-	-	-	-	Yes
	Piers, R. et al. (2021)	-	n.a.	+	+	-	+	-	Yes
	Ponsford, M. J. et al. (2021)	+		-	-	-	+	-	Yes
	Ramos-Rincon, J. M. Et al (2021)	+	n.a.	+	+	+	+	+	Yes
	Sablerolles, R. S. G. et al. (2021)	+	n.a.	+	+	+	+	+	Yes
	Tehrani, S. et al. (2021)	+	n.a.	+	+	-	+	-	Yes
	Thiam, C. N. et al. (2021)	-		-	-	-	+	-	Yes
	Osuafor C.N. et al. (2021)	+	n.a.	+	+	+	+	+	Yes
	Welch, C. et al. (2021)	-	-	-	+	-	+	-	Yes
	Wolfisberg, S. et al. (2021)	+	n.a.	?	+	-	+	-	Yes
HFRS	Kundi, H. et al. (2020)	+	n.a.	+	+	-	+	-	Yes
	Navaratnam, A. V. et al. (2021)	+	n.a.	+	+	-	+	-	Yes
	Ramos-Rincon, J. M. et al. (2021)	+	n.a.	+	+	+	+	+	Yes
MPI	Maki, Y. et al. (2021)	-	n.a.	-	-	-	-	-	Yes
	Pilotto, A. et al. (2021)	-	-	+	+	+	+	-	Yes
	Verholt, A. B. et al. (2021)	-	n.a.	?	+	?	+	-	Yes
mFI	Fumigalli, C. et al. (2021)	+	n.a.	?	+	-	+	-	No

B

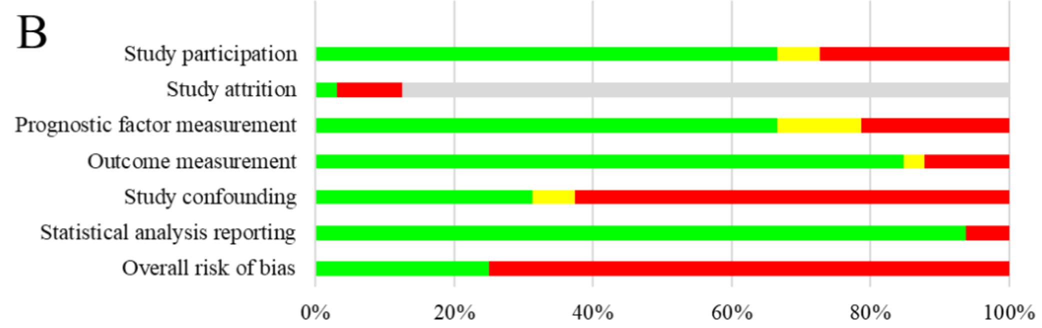

Figure S1 Risk of bias assessment on study level [A] and across studies [B] for studies reporting in-hospital mortality

For details please see the protocol for risk of bias assessment above.

Figure S2 — 30-day mortality

A

n.a. Not applicable
+ Low risk
? Moderate risk
- High risk

		Study participation	Study attrition	Prognostic factor measurement	Outcome measurement	Study confounding	Statistical analysis reporting	Overall risk of bias	Included in meta-analyses
CFS	Aliberti, M. J. R. et al. (2020)	+	n.a.	+	+	-	+	-	Yes
	Apea, V. J. et al. (2021)	+	n.a.	-	+	-	+	-	Yes
	Bielza, R. et al. (2021)	+	n.a.	?	+	-	+	-	Yes
	Davis, P. et al. (2020)	+	n.a.	+	+	-	+	-	Yes
	Dres, M. et al. (2021)	-	-	-	+	-	+	-	Yes
	Gilis, M. et al. (2020)	+	+	+	+	+	+	+	Yes
	Jung, C. et al. (2021)	+	+	+	+	-	+	-	Yes
	Maguire, D. et al. (2021)	+	n.a.	-	+	-	+	-	No
	Owen, R. K. et al. (2020)	?	n.a.	-	+	-	+	-	Yes
HFRS	Apea, V. J. et al. (2021)	+	n.a.	+	+	-	+	-	Yes
mFI	Kurtz, P. et al. (2021)	+	-	-	+	-	+	-	No

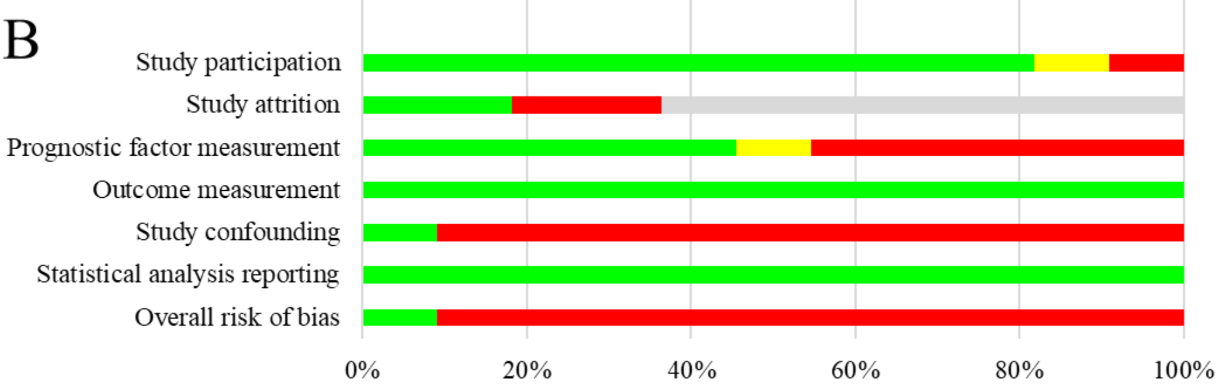

Figure S2 Risk of bias assessment on study level [A] and across studies [B] for studies reporting 30-day mortality

For details please see the protocol for risk of bias assessment above.

Figure S3 — Average frailty comparing deceased and discharged COVID-19 patients

A

n.a. Not applicable
+ Low risk
? Moderate risk
– High risk

	Study	Study participation	Study attrition	Prognostic factor measurement	Outcome measurement	Study confounding	Statistical analysis reporting	Overall risk of bias	Included in meta-analyses
CFS	Andrés-Esteban, E. M. et al. (2021)	-	n.a.	+	+	+	+	-	Yes
	Brill, S.E. et al. (2020)	+	n.a.	-	+	-	+	-	No
	Burns, G. P. et al. (2020)	?	n.a.	+	+	-	+	-	Yes
	Cecchini, S. et al. (2021)	+	n.a.	-	+	-	+	-	No
	Cuvelier, C. et al. (2021)	-	n.a.	-	-	-	-	-	No
	De Smet, R. et al. (2020)	+	n.a.	+	+	-	+	-	Yes
	Fagard, K. et al. (2021)	?	n.a.	+	+	-	+	-	Yes
	Hoek, R.A.S. et al. (2020)	?	n.a.	?	?	-	?	-	No
	Koduri, G. et. al. (2021)	+	n.a.	-	-	-	+	-	No
	Knights, H. et al. (2020)	+	n.a.	?	+	-	+	-	Yes
	Kundi, H. et al. (2020)	+	n.a.	+	+	-	+	-	No
	Mendes, A. et al. (2020)	+	n.a.	+	+	-	+	-	Yes
	Straw, S. et al. (2021)	+	n.a.	+	+	-	+	-	Yes
	van Steenkiste, J. et al. (2021)	+	n.a.	-	+	-	+	-	No
	Wolfisberg, S. et al. (2021)	+	n.a.	?	+	-	+	-	Yes

B

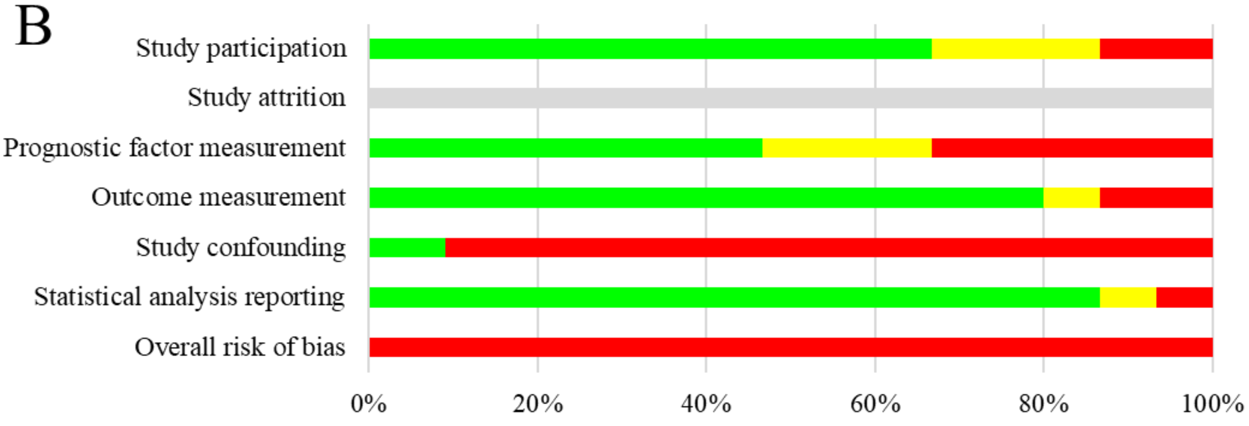

Figure S3 Risk of bias assessment on study level [A] and across studies [B] for studies reporting average frailty comparing discharged and deceased COVID-19 patients
For details please see the protocol for risk of bias assessment above.

Figure S4 — Average frailty comparing COVID-19 patients who survived for 30-days vs who did not survive

A	n.a. Not applicable<br>+ Low risk<br>? Moderate risk<br>- High risk	Study participation	Study attrition	Prognostic factor measurement	Outcome measurement	Study confounding	Statistical analysis reporting	Overall risk of bias	Included in meta-analyses
CFS	Baker, K. F. et al. (2021)	+	n.a.	-	+	-	+	-	Yes
	Bielza, R. et al. (2021)	+	n.a.	?	+	-	+	-	Yes
	Bradley, P. et al. (2020)	?	n.a.	+	+	-	+	-	Yes
	Fallon, A. et al. (2021)	-	n.a.	-	-	-	-	-	No
	Moledina, S. M. et al. (2020)	+	n.a.	?	+	-	+	-	Yes

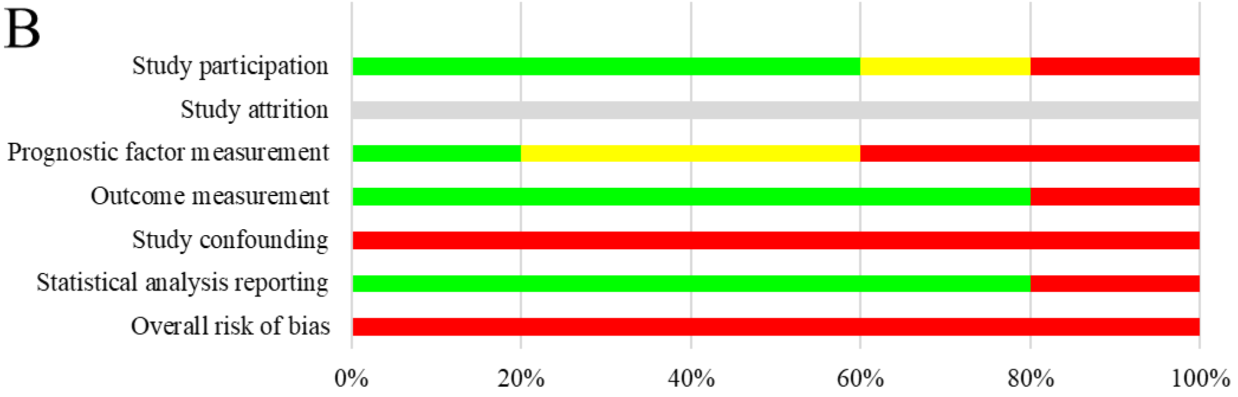

Figure S4 Risk of bias assessment on study level [A] and across studies [B] for studies reporting average frailty comparing COVID-19 patients who survived for 30 days and who did not
For details please see the protocol for risk of bias assessment above.

Figure S5 — ICU admission

A

n.a. Not applicable
+ Low risk
? Moderate risk
- High risk

		Study participation	Study attrition	Prognostic factor measurement	Outcome measurement	Study confounding	Statistical analysis reporting	Overall risk of bias	Included in meta-analyses
CFS	Andrés-Esteban, E. M. et al. (2021)	-	n.a.	+	-	+	+	-	Yes
	Aw, D. et al. (2020)	+	n.a.	+	-	+	+	-	Yes
	Blomaard, L. C. et al. (2021)	+	n.a.	-	-	?	+	-	Yes
	Gilis, M. et al. (2020)	+	+	+	-	+	+	-	Yes
	Marengoni, A. et al. (2020)	+	n.a.	+	+	-	+	-	No
	Osuafor C.N. et al. (2021)	+	n.a.	+	-	+	+	-	Yes
	Owen, R. K. et al. (2020)	?	n.a.	-	+	-	+	-	Yes
	Piers, R. et al. (2021)	-	n.a.	+	-	-	+	-	Yes
	Ramos-Rincon, J. M. et al. (2021)	+	n.a.	+	+	+	+	+	Yes
	Sablerolles, R. S. G. et al. (2021)	+	n.a.	+	+	-	+	-	Yes
	Wolfisberg, S. et al. (2021)	+	n.a.	?	+	-	+	-	Yes

B

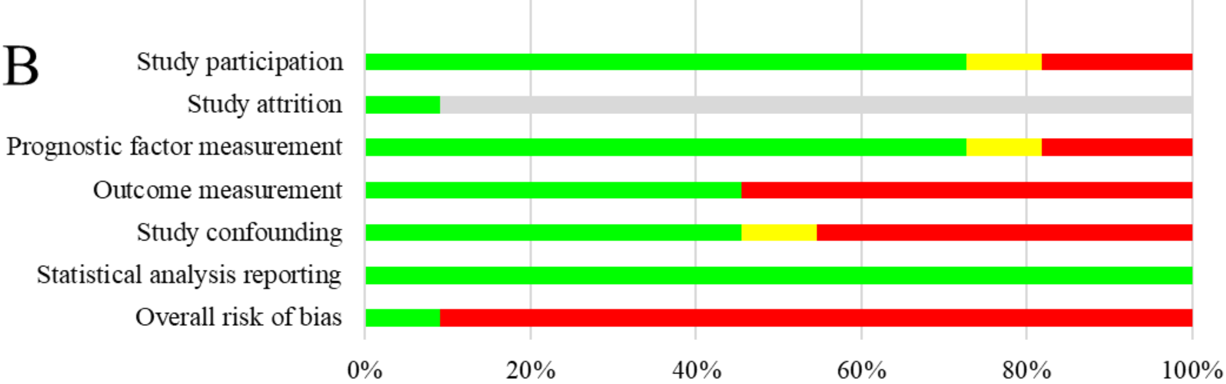

Figure S5 Risk of bias assessment on study level [A] and across studies [B] reporting intensive care admission

For details, please see the protocol for risk of bias assessment above.

Figure S6 — Length of hospital stay

A

n.a. Not applicable
+ Low risk
? Moderate risk
- High risk

		Study participation	Study attrition	Prognostic factor measurement	Outcome measurement	Study confounding	Statistical analysis reporting	Overall risk of bias	Included in meta-analyses
CFS	Andrés-Esteban, E. M. et al. (2021)	-	n.a.	+	+	+	+	-	No
	Bavaro, D. F. et al. (2021)	+	n.a.	?	-	+	+	-	No
	Blomaard, L. C. et al. (2021)	+	n.a.	-	+	?	+	-	No
	Hewitt, J. et al. (2020)	+	+	+	+	-	+	-	No
	Kundi, H. et al. (2020)	+	n.a.	+	+	-	+	-	No
	Osuafor C.N. et al. (2021)	+	n.a.	?	+	+	+	+	No
	Ramos-Rincon, J. M. et al. (2021)	+	n.a.	+	+	+	+	+	No
HFRS	Ramos-Rincon, J. M. et al. (2021)	+	n.a.	+	+	+	+	+	No

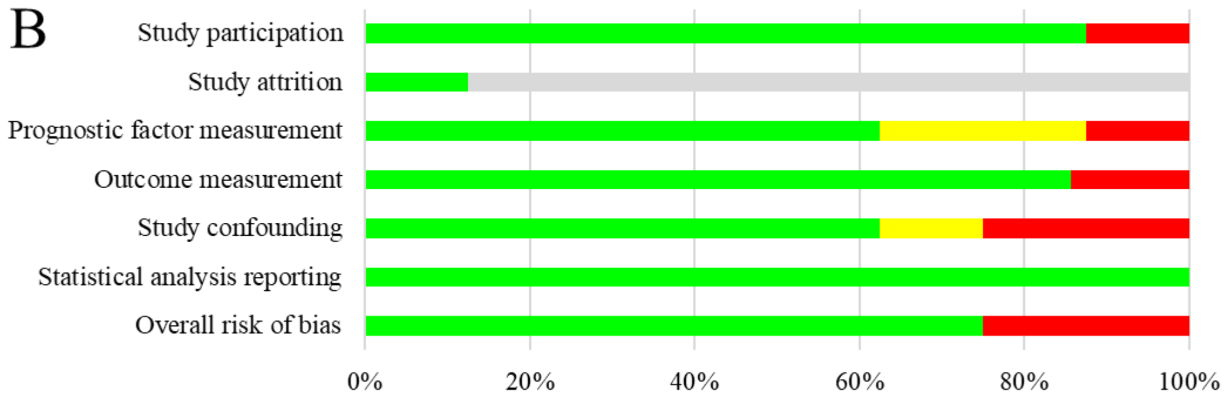

Figure S6 Risk of bias assessment on study level [A] and across studies [B] reporting length of stay
For details, please see the protocol for risk of bias assessment above.

Mortality in patients with CFS 1–3 vs 4–9

Figure S7 – Forest plot for mortality grouped by follow-up

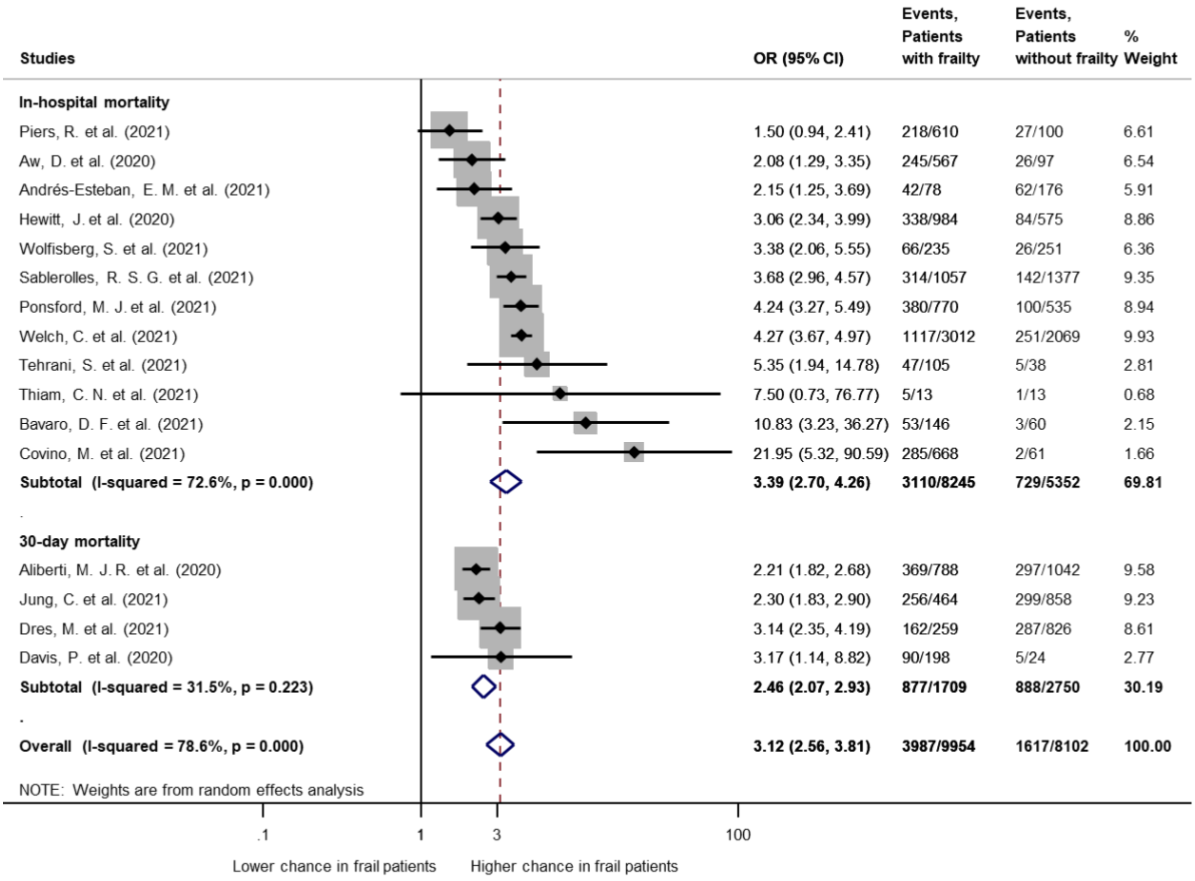

Figure S7 30-day and in-hospital mortality in patients with frailty indicated by CFS

Frail patients (CFS 4–9) have significantly higher odds of in-hospital (OR: 3.39; CI: 2.70–4.26) and 30-day mortality (OR: 2.46; CI: 2.07–2.93), the overall odds ratio being 3.12 (CI: 2.56–3.81). Note that heterogeneity was not significant for 30-day mortality, but significant for in-hospital mortality and in overall. OR: odds ratio; CI: confidence interval. P>0.1 was considered significant.

Figure S8 – Forest plot grouped by age restriction

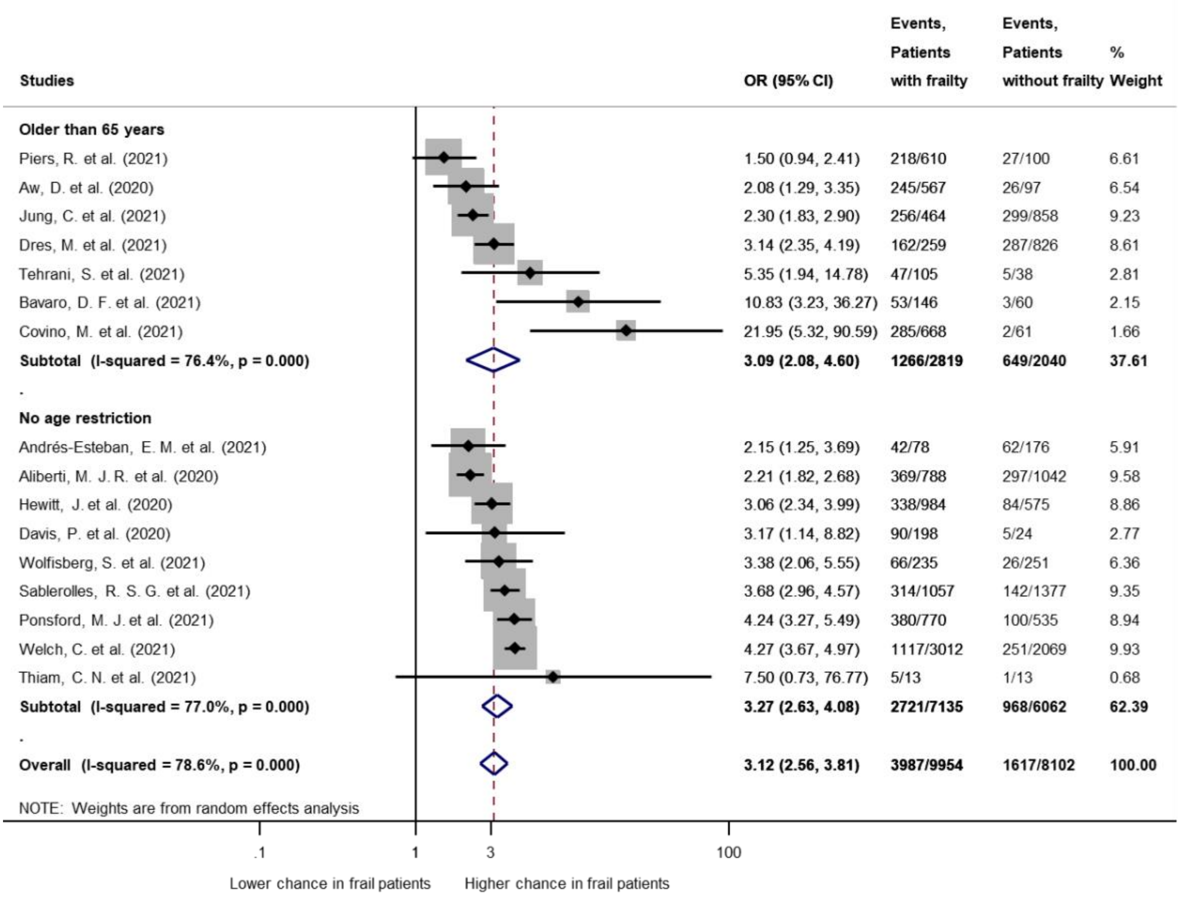

Figure S8 Mortality in frail patients indicated by CFS (1–3 vs 4–9), with studies grouped by age restriction

Studies only enrolling patients older than 65 years of age have an overall odds ratio of 3.09 (CI: 2.08–4.60) for mortality in frail patients (CFS 5–9) while studies without age restriction have an overall OR of 3.27 (CI: 2.63–4.08). Note that heterogeneity was significant in both subgroups and for the overall results as well. OR: odds ratio; CI: confidence interval. P<0.1 was considered significant.

Figure S9 – Leave-one-out analysis

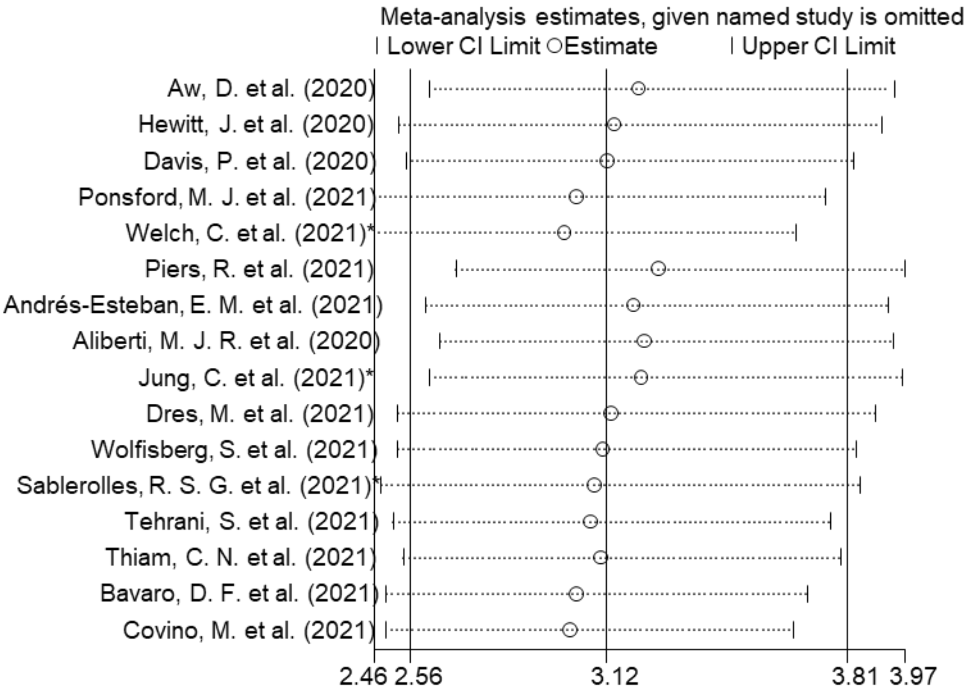

Figure S9 Leave-one-out sensitivity analysis for studies reporting mortality in patients with CFS 1–3 vs CFS 4–9

Each row shows the overall OR and CI with the omission of the indicated study. There is no study the omission of which would change statistical significance.

Figure S10 – Funnel plot

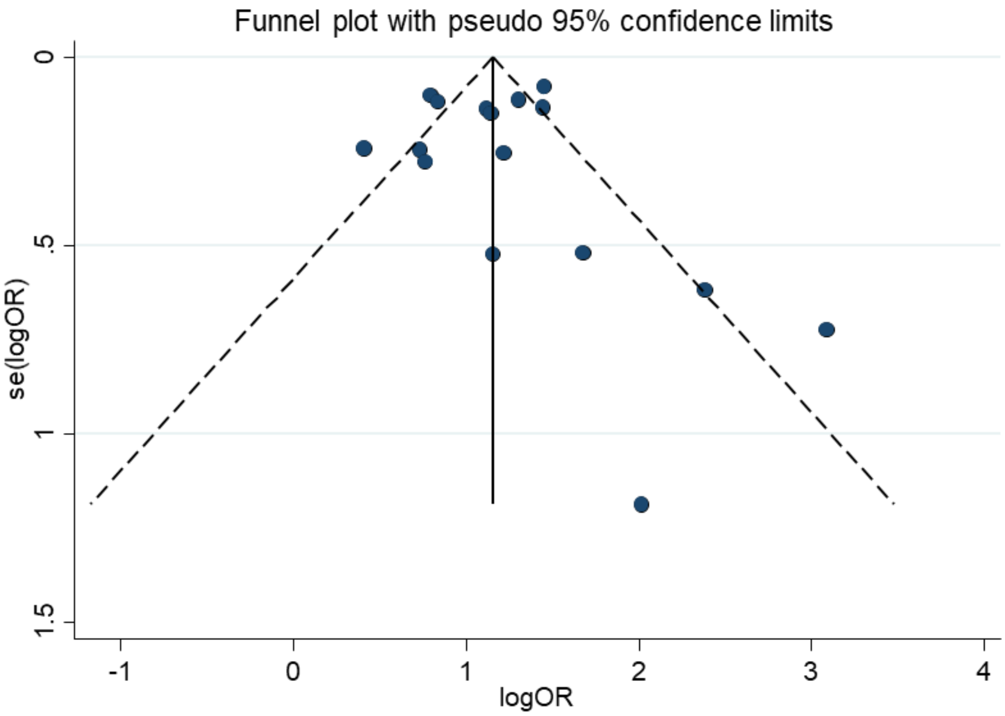

Figure S10 Funnel plot for mortality in patients with CFS 1–3 vs 4–9
Based on the visual inspection of the funnel plot and the result of the Eggers' test (p=0.858) no small study effect was identified.

Mortality in patients with CFS 1–4 vs 5–9

Figure S11 — Forest plot for mortality grouped by follow-up

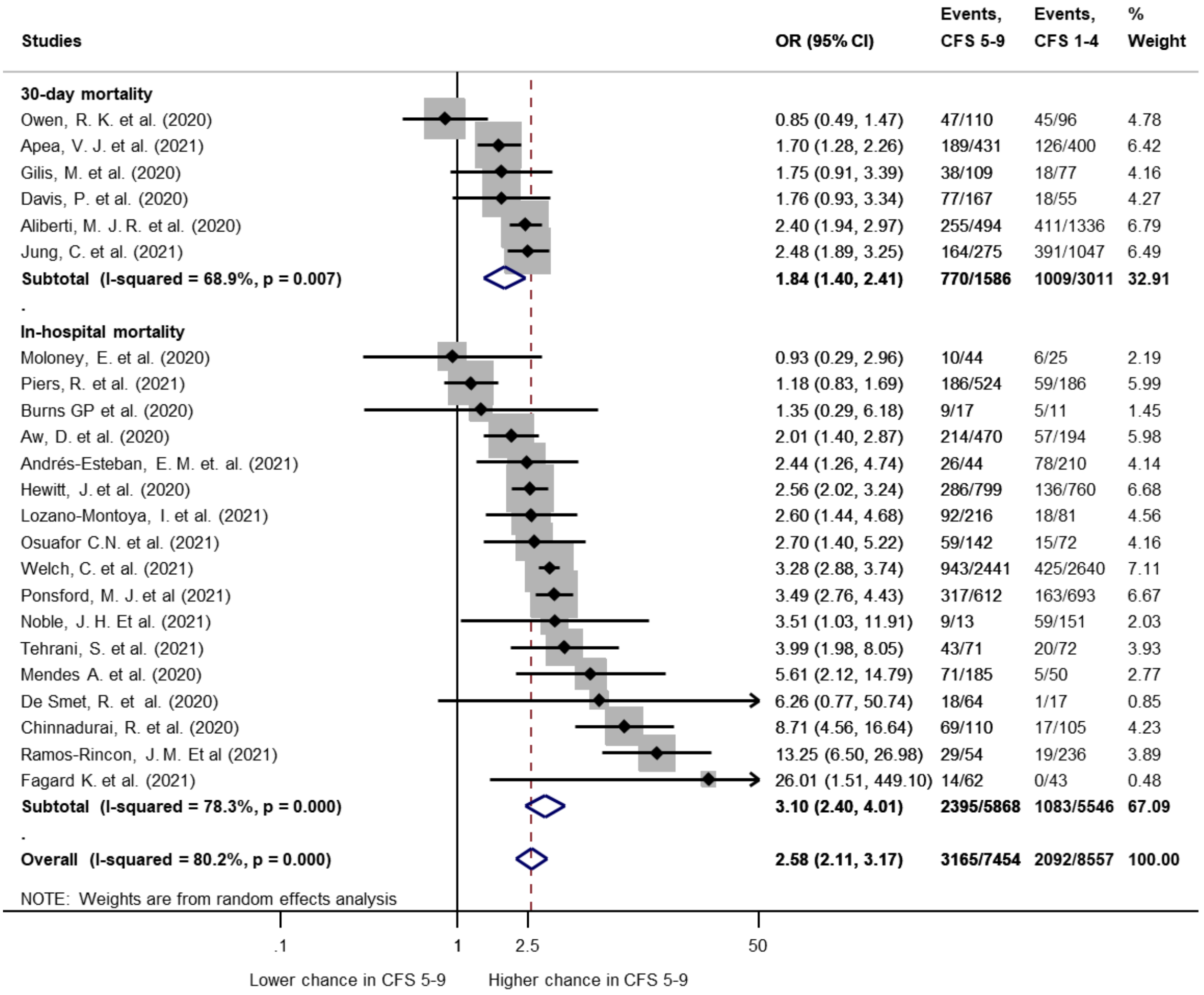

Figure S11 30-day and in-hospital mortality in patients with CFS 1–4 vs 5–9
Patients with CFS 4–9 have significantly higher odds of 30-day mortality (OR: 1.84; CI: 1.40–2.41) and in-hospital mortality (OR: 3.10; CI: 2.40–4.01), the overall odds ratio being 2.58 (CI: 2.11–3.17). Note that heterogeneity was significant in both subgroups. OR: odds ratio; CI: confidence interval. P>0.1 was considered significant.

Figure S12 — Forest plot grouped by age restriction

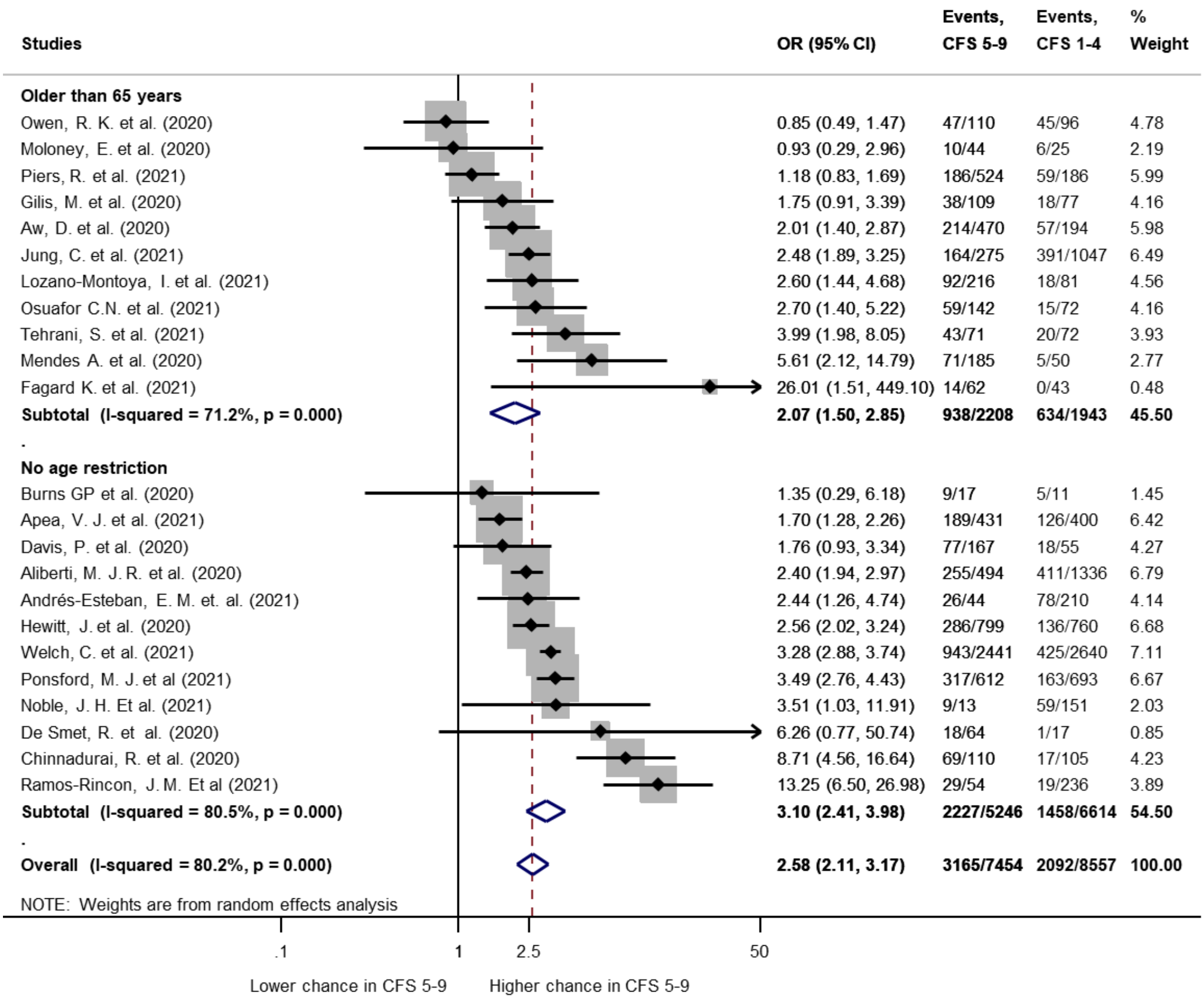

Figure S12 Mortality in patients with CFS 1–4 vs 5–9, with studies grouped by age restriction
Studies only enrolling patients older than 65 years of age have an overall odds ratio of 2.07 (CI: 1.50–2.85) for mortality in patients CFS 5–9 while studies without age restriction have an overall OR of 3.10 (CI: 2.11–3.17). Note that heterogeneity was significant in both subgroups and for the overall results as well. OR: odds ratio; CI: confidence interval. P<0.1 was considered significant.

Figure S13 – Leave-one-out analysis

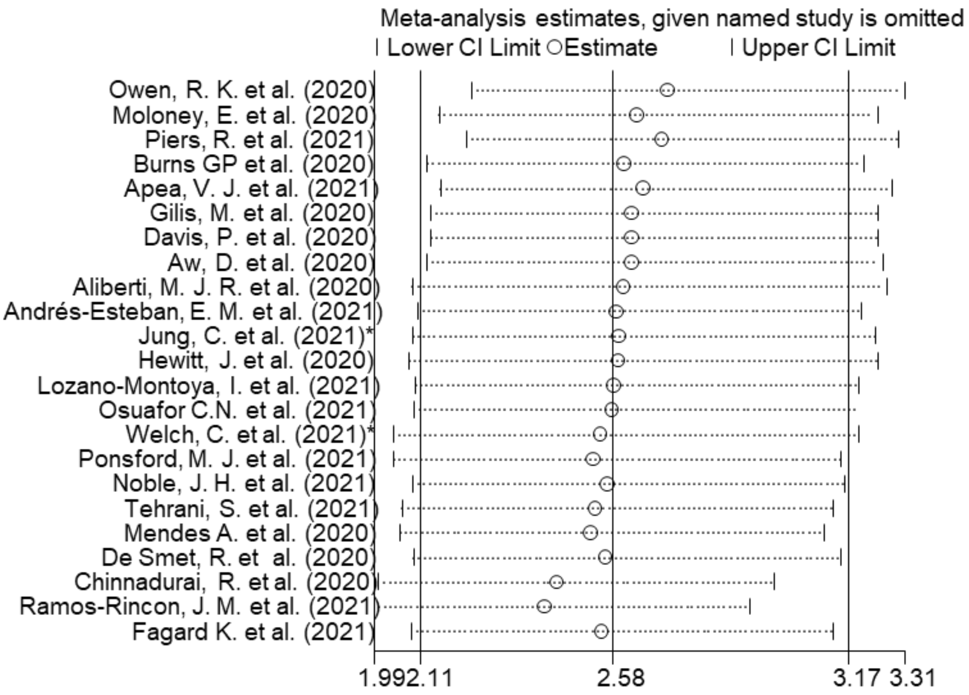

Figure S13 Leave-one-out sensitivity analysis for studies reporting mortality in patients with CFS 1–4 vs CFS 5–9

Each row shows the overall OR and CI with the omission of the indicated study. There is no study the omission of which would change statistical significance.

Figure S14 – Funnel plot

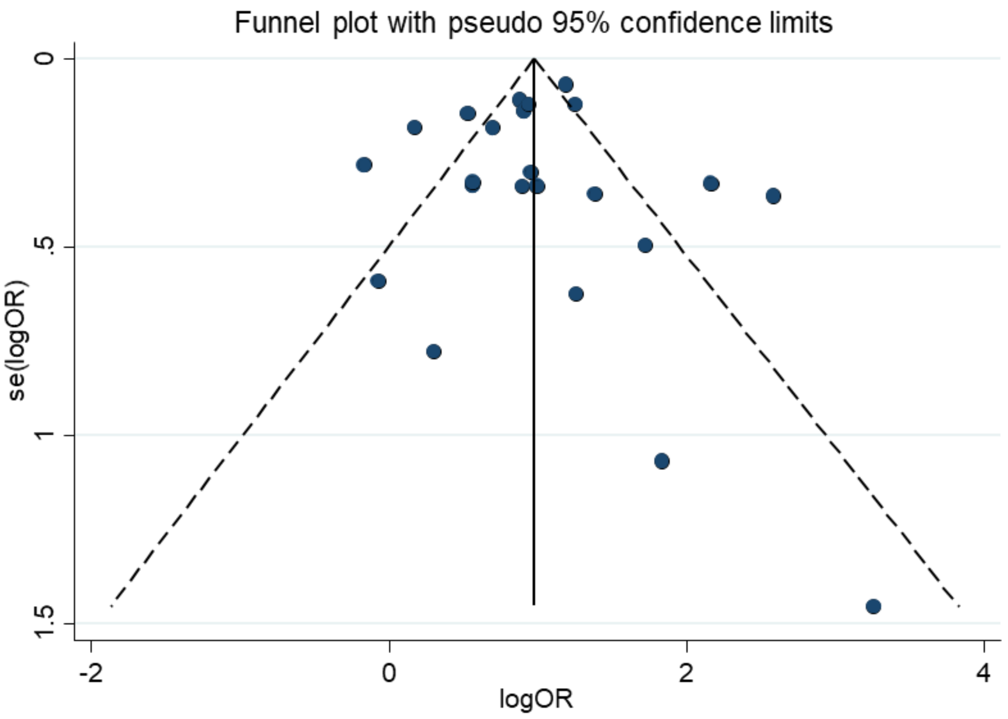

Figure S14 Funnel plot for mortality in patients with CFS 1–4 vs 5–9
Based on the visual inspection of the funnel plot and the result of the Eggers' test (p=0.813) no small study effect was identified.

Mortality in patients with CFS 1–5 vs 6–9

Figure S15 – Forest plot, studies grouped by country

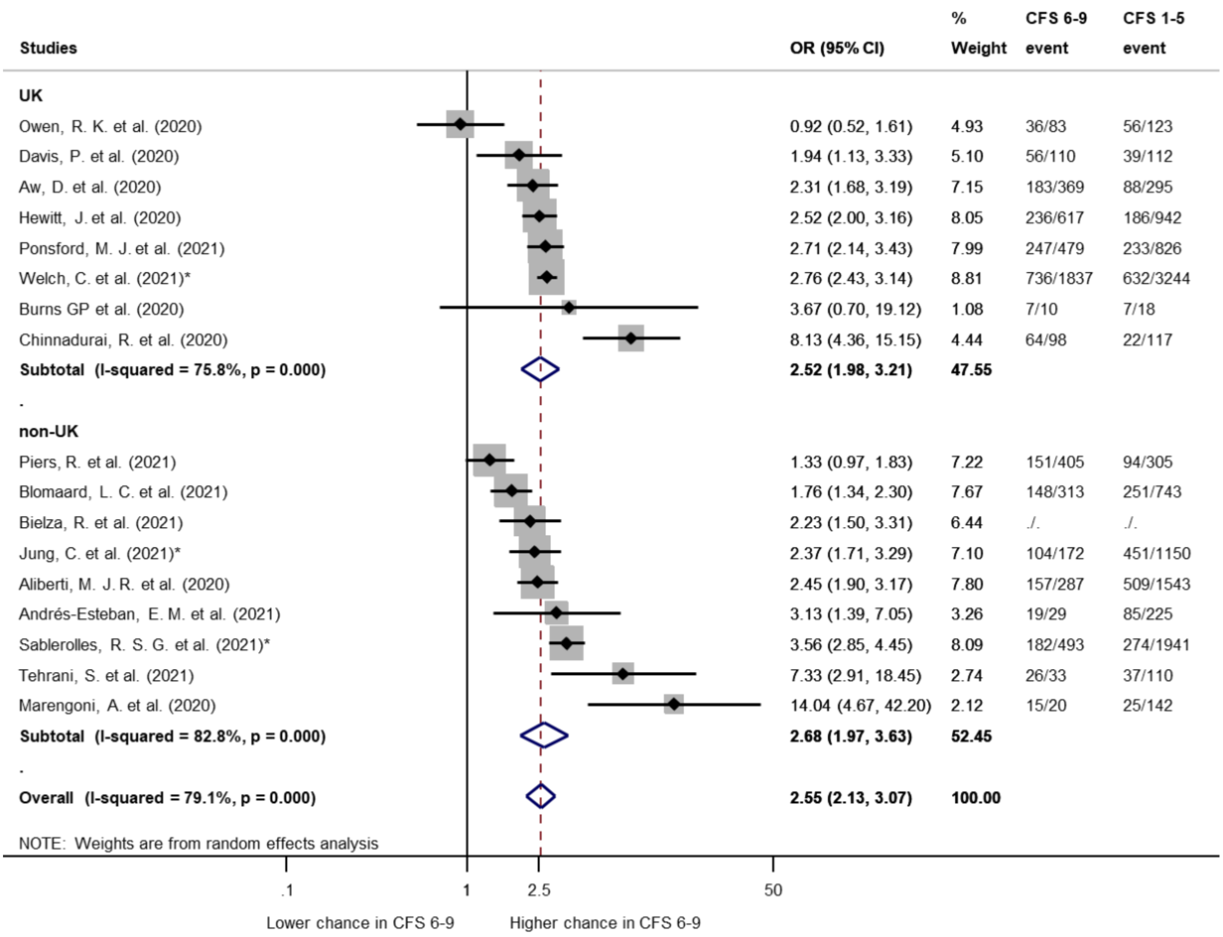

Figure S15 Mortality comparing CFS 1–5 and CFS 6–9 groups, with studies grouped by country

Studies from the UK have an overall odds ratio of 2.52 (CI: 1.98–3.21) for mortality in patients with CFS 6–9 while studies outside the UK (non-UK) showed a similar subtotal OR of 2.55 (CI: 1.97–3.63). Note that heterogeneity was significant in both subgroups and for the overall results as well. OR: odds ratio; CI: confidence interval. P<0.1 was considered significant.

Figure S16 — Forest plot for mortality grouped by follow-up

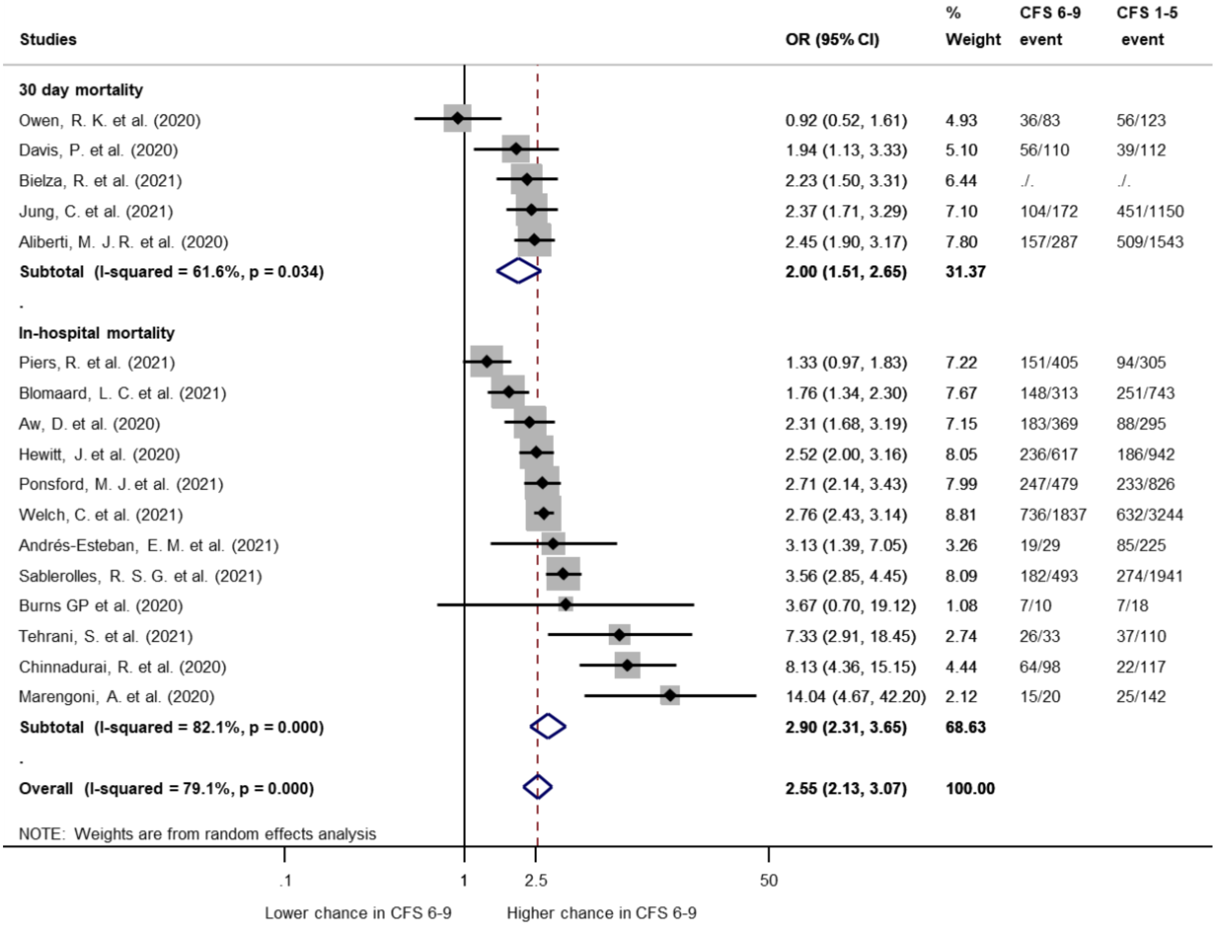

Figure S16 30-day and in-hospital mortality in patients with CFS 1–5 vs 6–9

Patients with CFS 4–9 have significantly higher odds of 30-day mortality (OR: 2.00; CI: 1.51–2.65) and in-hospital mortality (OR: 2.90; CI: 2.31–3.65), the overall odds ratio being 2.55 (CI: 2.13–3.07). Note that heterogeneity was significant in both subgroups. OR: odds ratio; CI: confidence interval. P<0.1 was considered significant.

Figure S17 — Forest plot grouped by age restriction

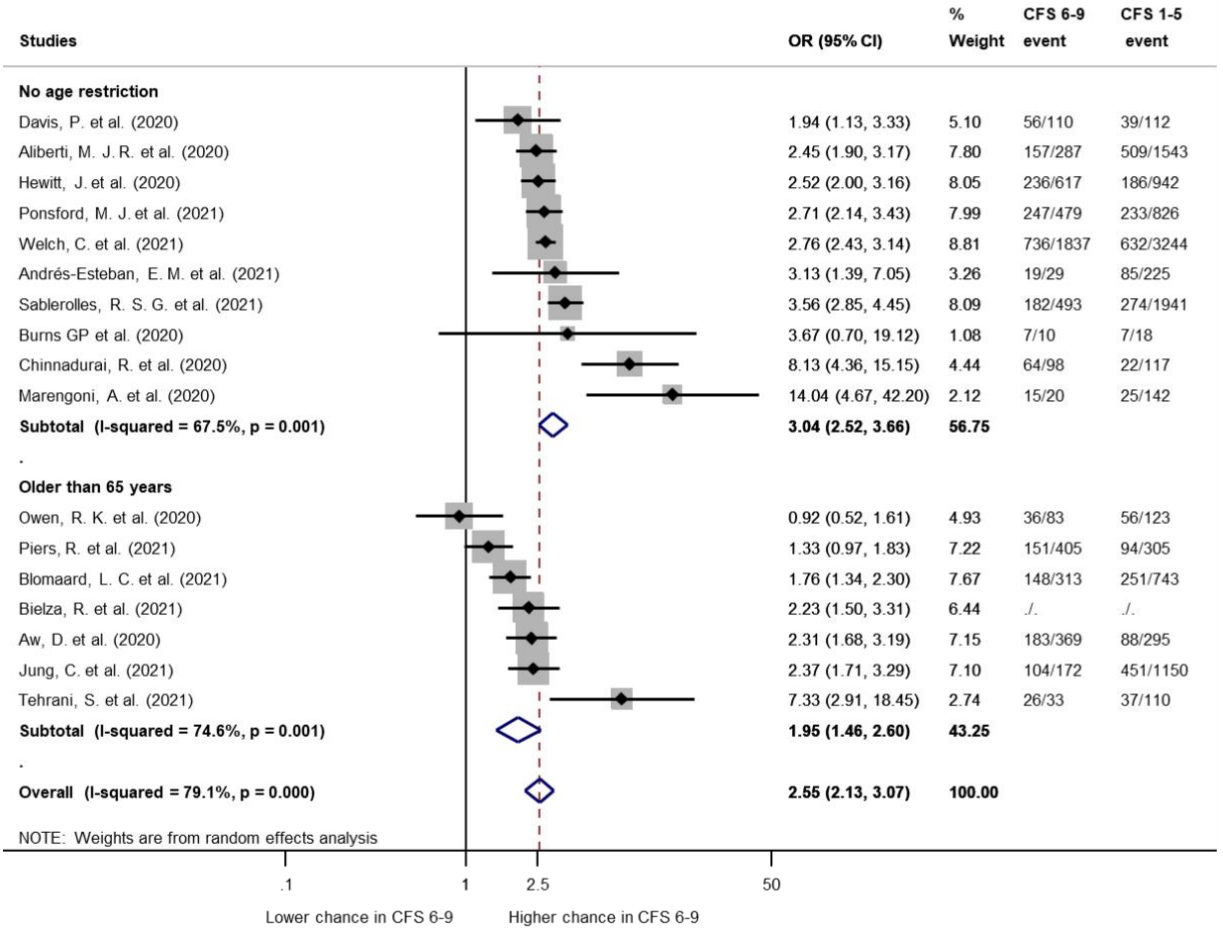

Figure S17 Mortality comparing CFS 1–5 and CFS 6–9 groups, with studies grouped by age restriction

Studies only enrolling patients older than 65 years of age have an overall odds ratio of 1.95 (CI: 1.46–2.60) for mortality in patients with CFS 6–9 while studies without age restriction have an overall OR of 3.04 (CI: 2.52–3.66). Note that heterogeneity was significant in both subgroups and for the overall result as well. OR: odds ratio; CI: confidence interval. P<0.1 was considered significant.

Figure S18 — Leave-one-out analysis

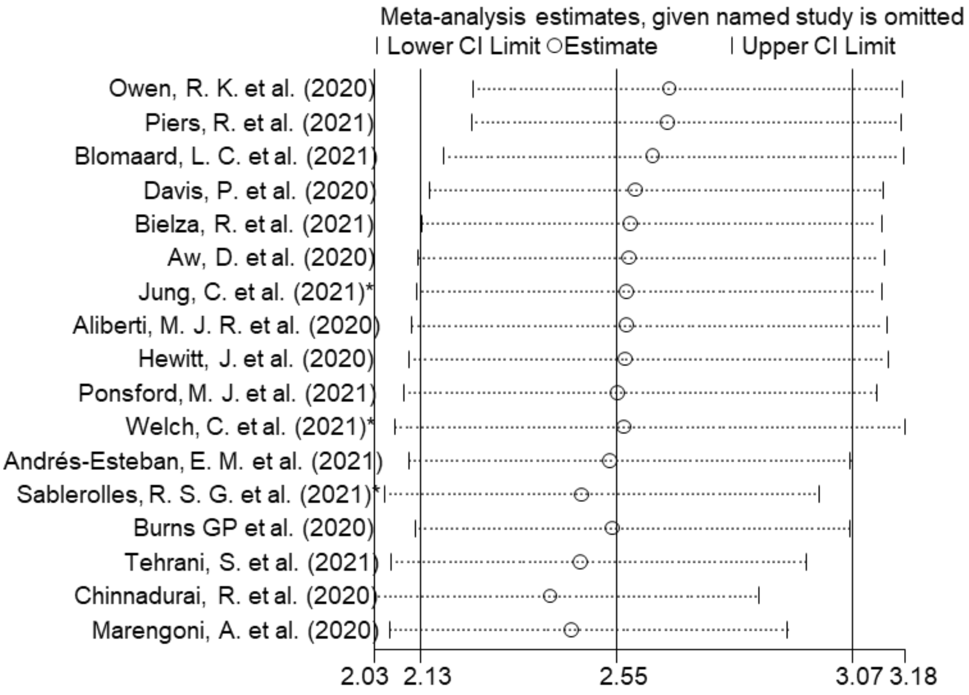

Figure S18 Leave-one-out sensitivity analysis for studies reporting mortality in patients with CFS 1–5 vs CFS 6–9

Each row shows the overall OR and CI with the omission of the indicated study. There is no study the omission of which would change statistical significance.

Figure S19 – Funnel plot

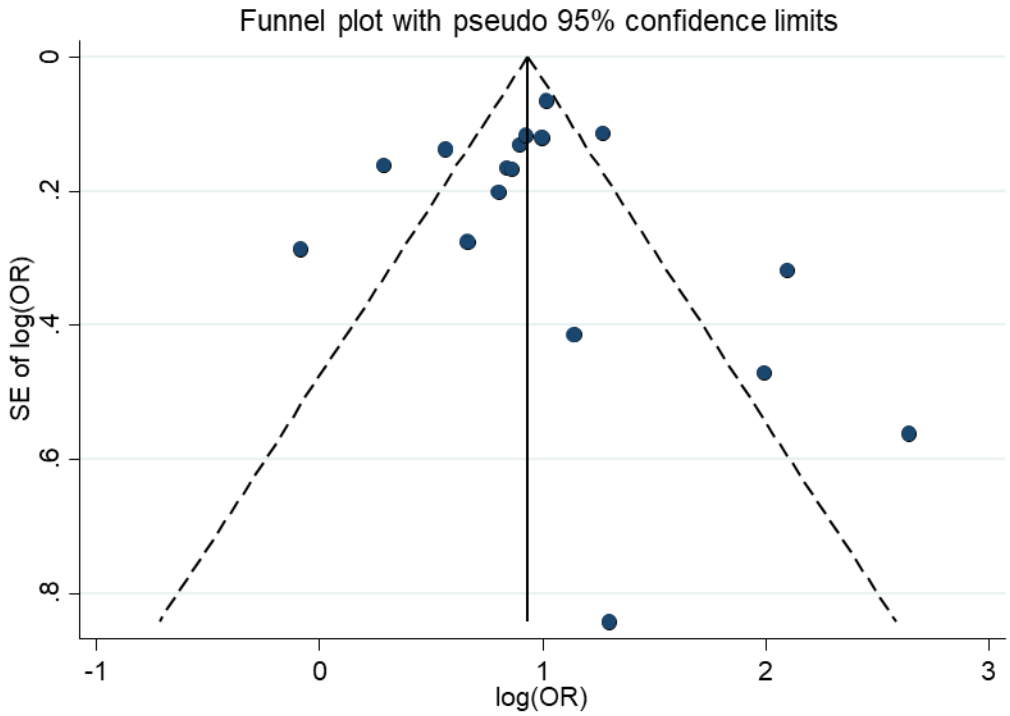

Figure S19 Funnel plot for mortality in patients with CFS 1–5 vs 6–9
Based on the visual inspection of the funnel plot and the result of the Eggers' test (p=0.792) no small study effect was identified.

Weighted Mean Difference of CFS

Figure S20 – Forest plot, studies grouped by country

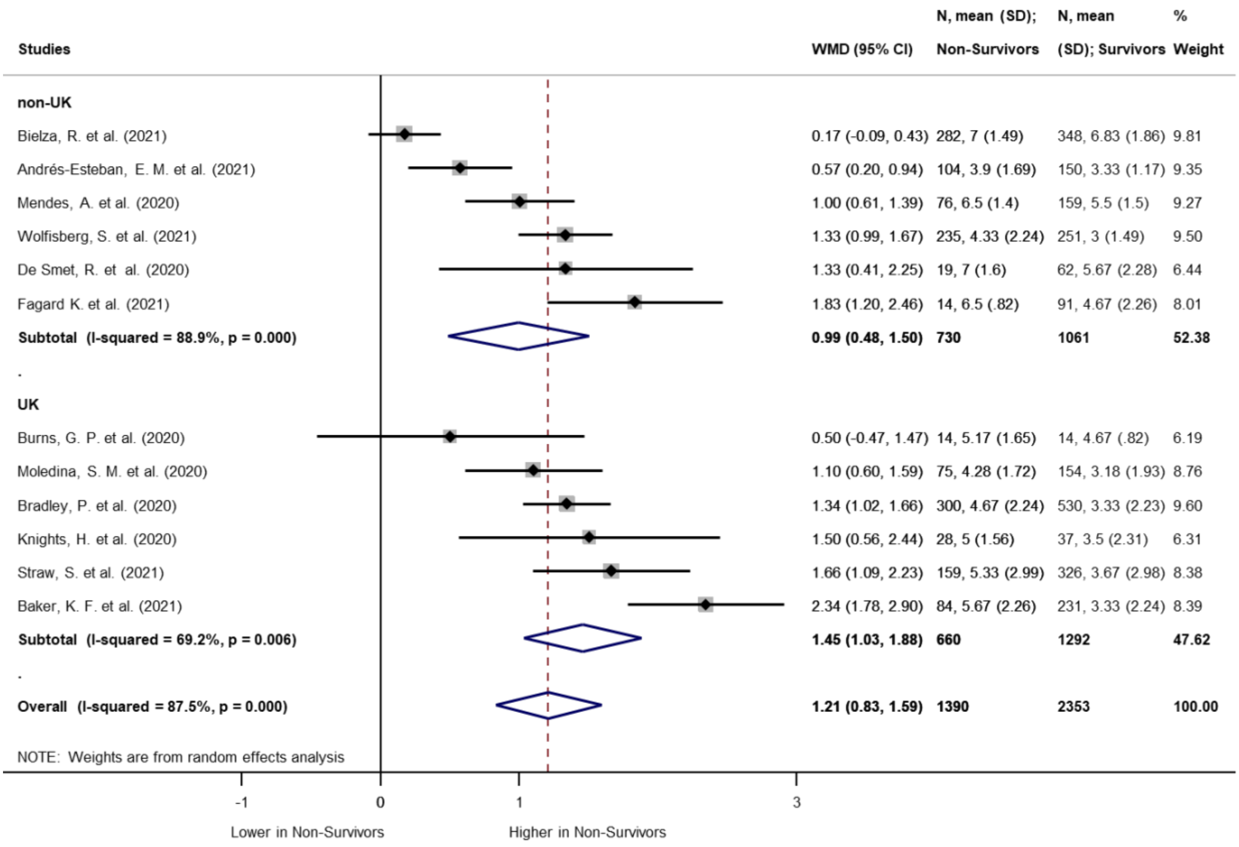

Figure S20 Weighted mean difference of CFS score for mortality with studies grouped by country
Non-survivors average CFS scores were significantly higher than survivors' in both the UK and the non-UK subgroup (WMD: 0.99; CI: 0.48–1.50 for non-UK and WMD: 1.45; CI: 1.03–1.88 for UK subgroup) Please note the significant heterogeneity in both subgroups and for the overall result. WMD: weighted mean difference; SD: standard deviation; CI: confidence interval. P<0.1 was considered significant.

Figure S21 – Forest plot for mortality grouped by follow-up

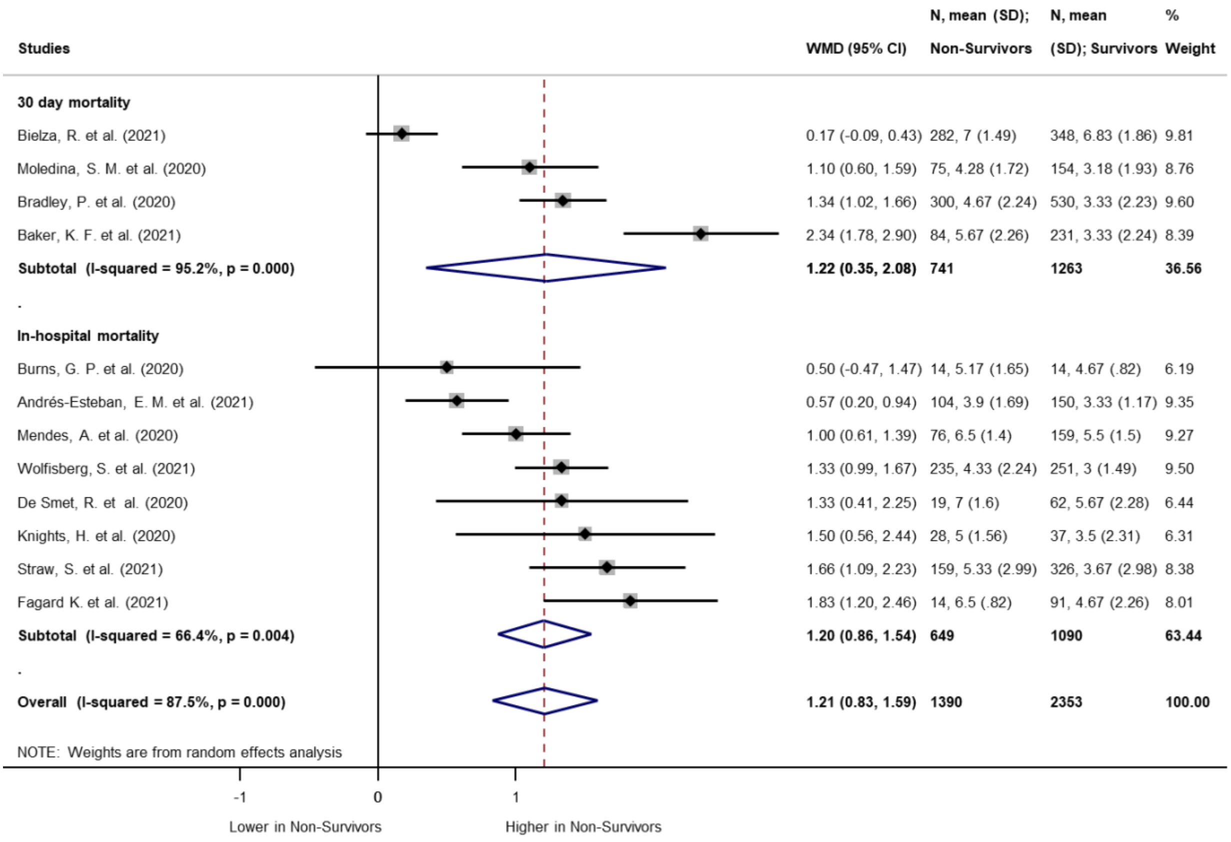

Figure S21 Weighted mean difference of CFS score for mortality with studies grouped by follow-up
Non-survivors average CFS scores were significantly higher than survivors' in both the 30-day and the in-hospital mortality subgroup (WMD: 1.22; CI: 0.35–2.08 for 30-day and WMD: 1.20; CI: 0.86–1.54 for in-hospital mortality subgroup) Please note the significant heterogeneity in both subgroups and for the overall result. WMD: weighted mean difference; SD: standard deviation; CI: confidence interval. P<0.1 was considered significant.

Figure S22 — Leave-one-out analysis

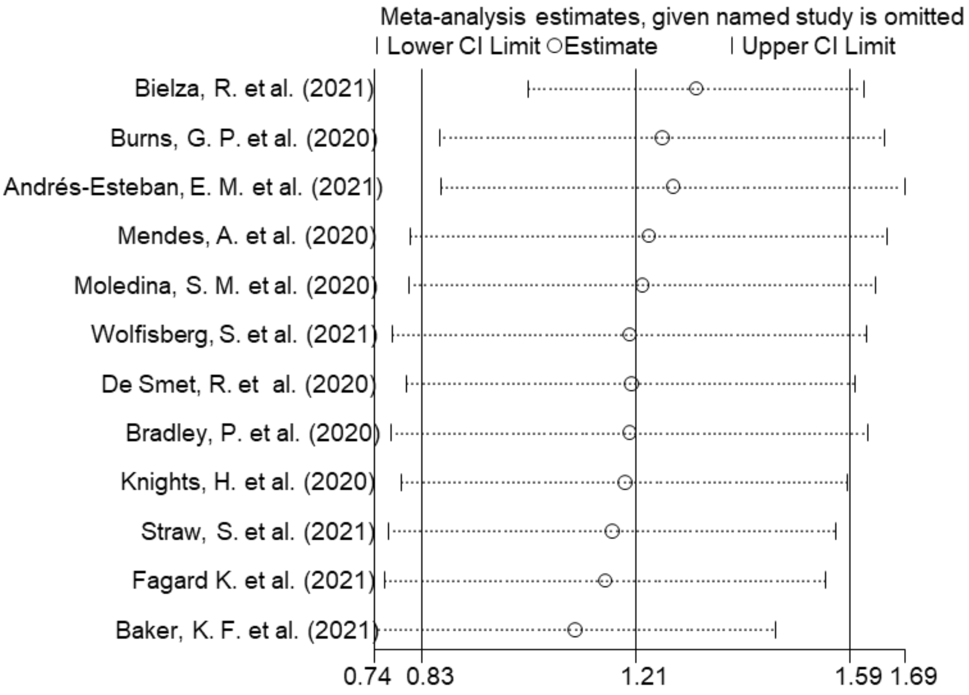

Figure S22 Leave-one-out sensitivity analysis for studies reporting average frailty indicated by CFS in survivors and non-survivors

Each row shows the overall OR and CI with the omission of the indicated study. There is no study the omission of which would change statistical significance.

Figure S23 – Funnel plot

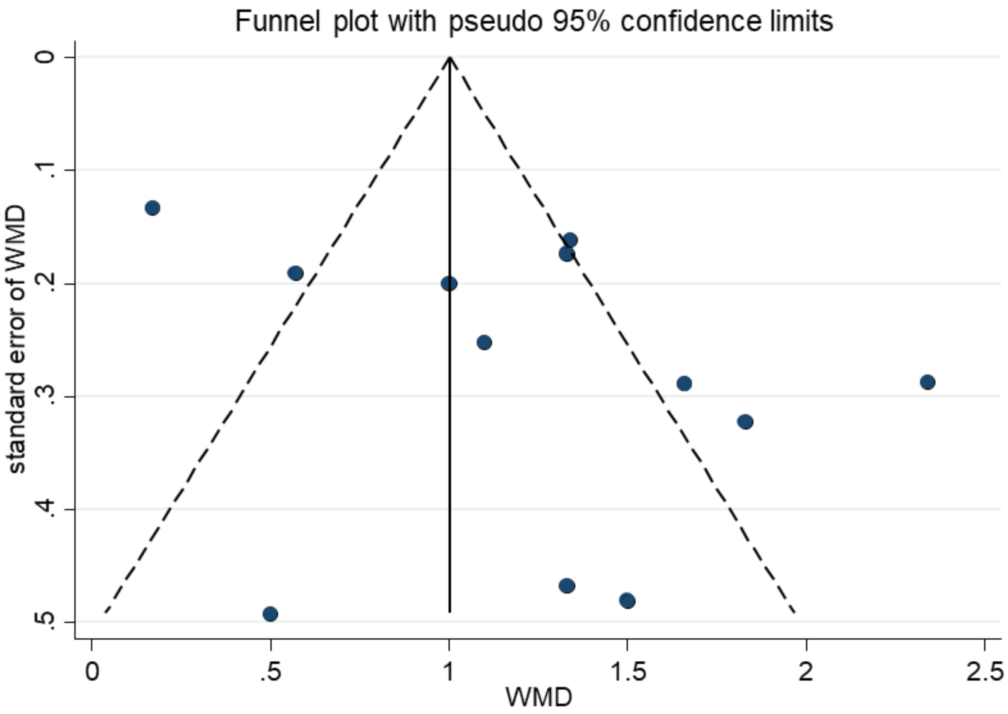

Figure S23 Funnel plot for frailty difference in survivors vs non-survivors
Based on the visual inspection of the funnel plot and the result of the Eggers' test (p=0.108) no small study effect was identified.

Mortality in patients with MPI 1 vs 2+3

Figure S24 – Forest plot

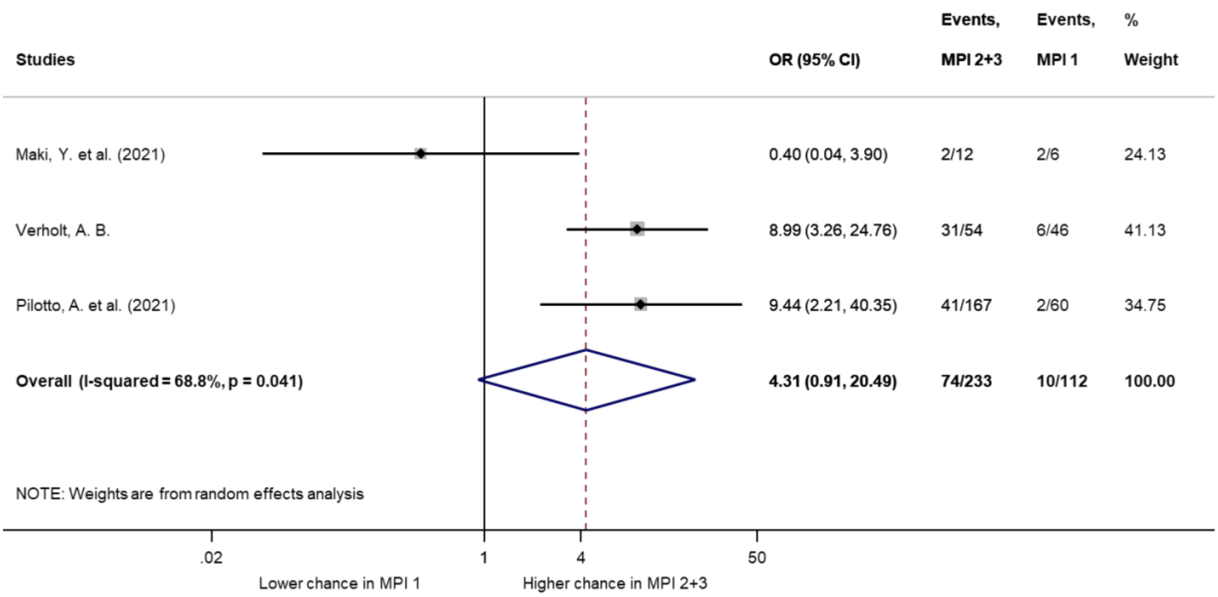

Figure S24 Mortality in patients with MPI 1 vs 2 and 3

Patients with in the MPI 2 and 3 category have an overall odds ratio of 4.31 (CI: 0.91–20.49) for mortality compared to patients in the MPI 1 category. Note that heterogeneity was significant. OR: odds ratio; CI: confidence interval. P<0.1 was considered significant.

ICU admission in patients with CFS 1–3 vs 4–9

Figure S25 – Leave-one-out analysis

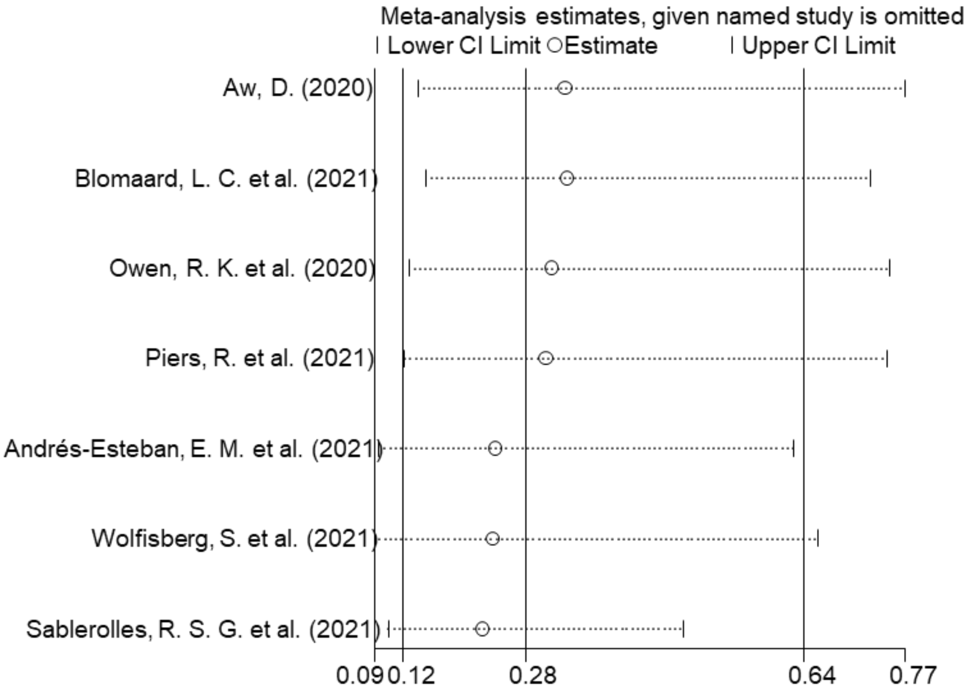

Figure S25 Leave-one-out sensitivity analysis for studies reporting ICU admission in patients with CFS 1–3 vs CFS 4–9

Each row shows the overall OR and CI with the omission of the indicated study. There is no study the omission of which would change statistical significance.

Figure S26 – Funnel plot

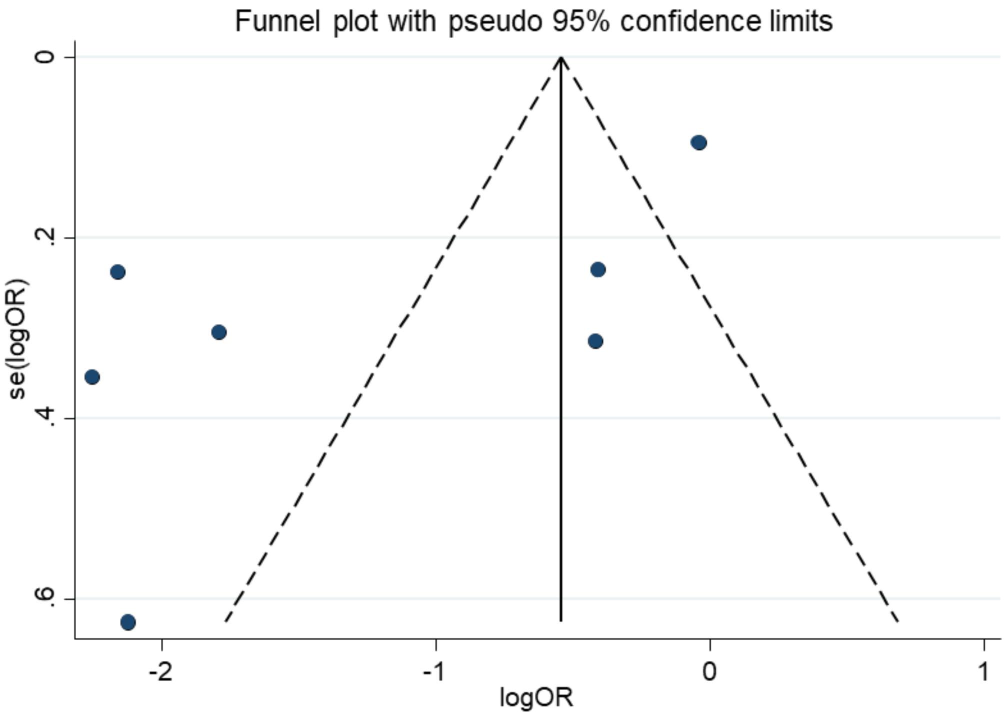

Figure S25 Funnel plot for ICU admission in patients with CFS 1–3 vs 4–9
Visual inspection raises the suspicion of small-study effect. Egger's test was not conducted due to the low number of studies.

ICU admission in patients with CFS 1–4 vs 5–9

Figure S27 – Forest plot

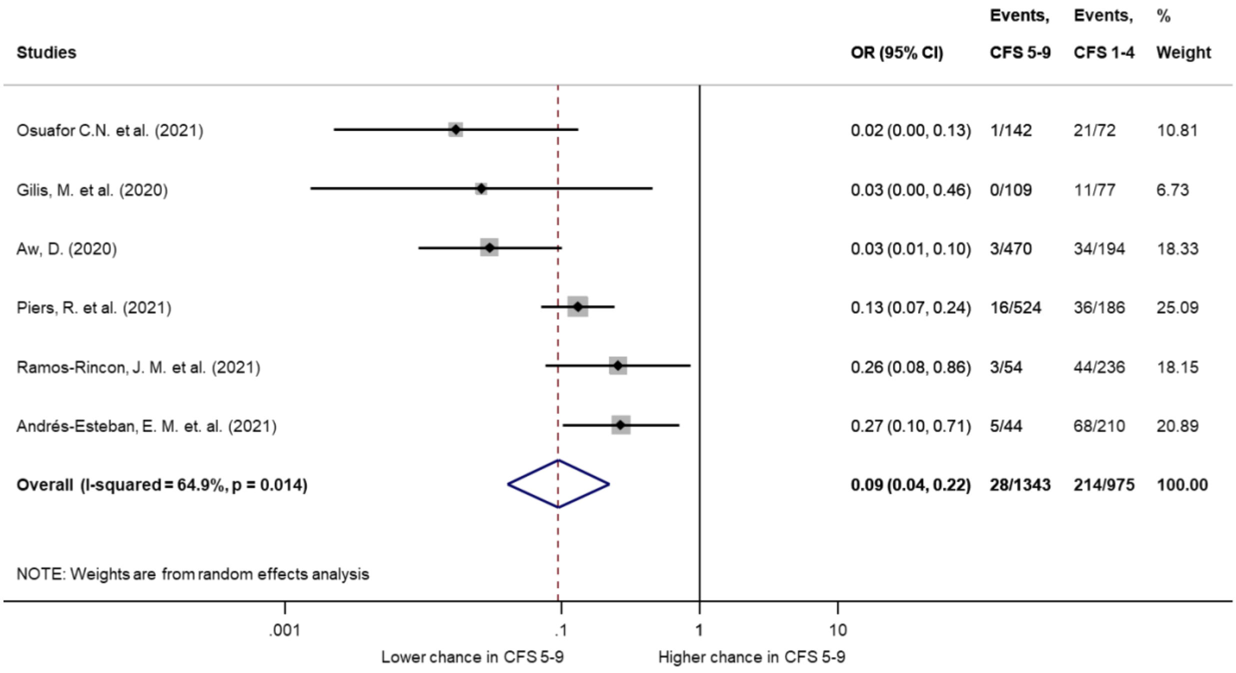

Figure S27 ICU admission in patients with CFS 1–4 vs 5–9

COVID-19 patients with CFS 5-9 have an overall OR of 0.09 (CI: 0.04–0.22). Note significant heterogeneity. OR: odds ratio; CI: confidence interval. P<0.1 was considered significant.

Figure S28 – Leave-one-out analysis

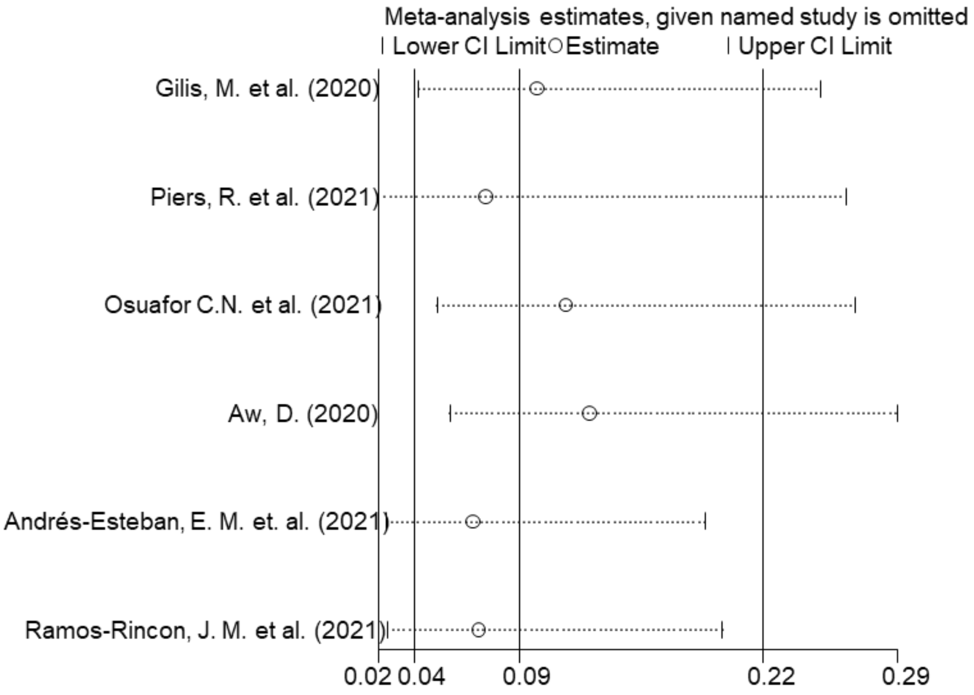

Figure S28 Leave-one-out sensitivity analysis for studies reporting ICU admission in patients with CFS 1–4 vs CFS 5–9

Each row shows the overall OR and CI with the omission of the indicated study. There is no study the omission of which would change statistical significance.

Figure S29 – Funnel plot

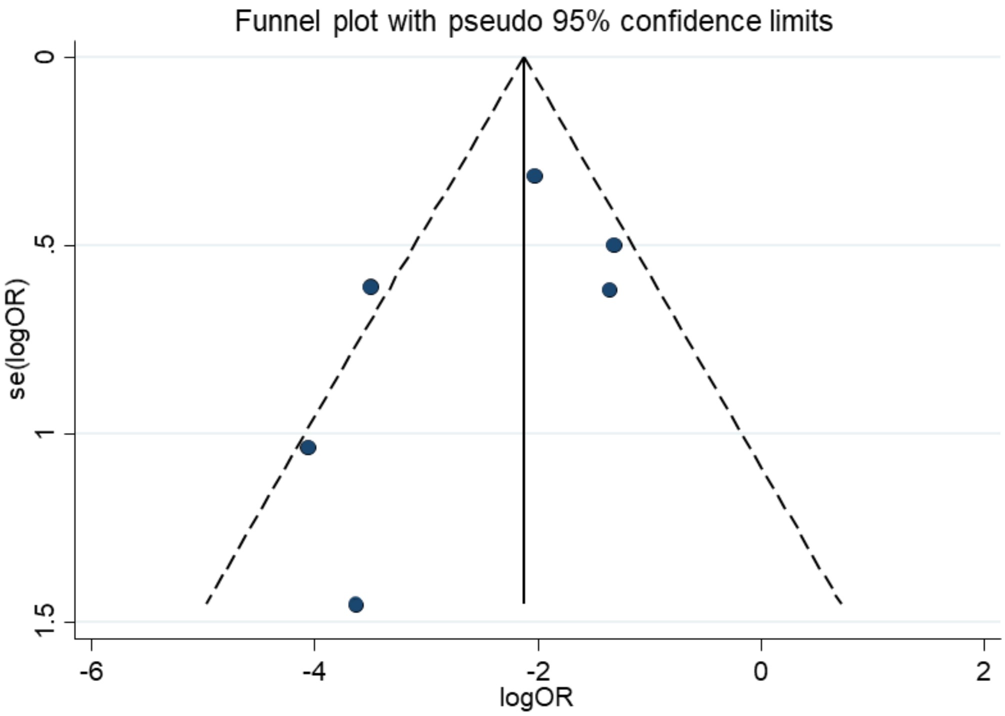

Figure S29 Funnel plot for ICU admission in patients with CFS 1–4 vs 5–9
Visual inspection does not raise the suspicion of small-study effect. Egger's test was not conducted due to the low number of studies.

Length of hospital stay

Summary of reported results

**Andrés-Esteban**	**CFS**	**1 – 3**	**4**	**5 – 9**	
	median (IQR)	11 (9 – 19)	10 (7 – 14)	5 (1 – 11)	
**Bavaro**	**CFS**	**1 – 3**	**4 – 6**	**7 – 9**	
	median (IQR)	22 (15 – 42)	25 (14 – 37)	21 (7 – 37)	
**Blomaard**	**CFS**	**1 – 3**	**4 – 5**	**6 – 9**	
	median (IQR)	6 (3 – 11)	6 (3 – 10)	6 (3 – 10)	
**Hewitt**	**CFS**	**1 – 2**	**3 – 4**	**5 – 6**	**7 – 9**
	Crude HR (95% CI)	1	0.87 (0.71 – 1.05)	0.61 (0.49 – 0.76)	0.56 (0.44 – 0.72)
**Kundi**	**LOS > 10 days HFRS**	**<5**	**5–15**	**>15**	
	Adjusted OR (95% CI)	1.00	1.152 (1.067 – 1.243)	1.317 (1.169 – 1.483)	
**Osuafor**	**LOS > 10 days CFS**	**All**	**1 – 4**	**5 – 8**	
	n / all (%)	114 / 214 (53.5)	32 / 72 (45.1)	82 / 142 (57.8)	
**Ramos-Rincon**	**CFS LOS > 10 days**	**all**	**1 – 4**	**5 – 6**	**7 – 9**
	n / all (%)	131 / 290 (45.2)	109 / 236 (46.2)	15 / 33 (45.5)	7 / 21 (33.3)
	**HFRS LOS > 10 days**	**all**	**> 5**	**5 – 15**	**> 15**
	n / all (%)	131 / 290 (45.2)	89 / 225 (39.6)	31 / 49 (63.3)	11 / 16 (68.8)

References

1. Hayden JA, Côté P, Bombardier C: **Evaluation of the Quality of Prognosis Studies in Systematic Reviews**. *Annals of Internal Medicine* 2006, **144**(6):427-437.
2. Grooten WJA, Tseli E, Äng BO, Boersma K, Stålnacke B-M, Gerdle B, Enthoven P: **Elaborating on the assessment of the risk of bias in prognostic studies in pain rehabilitation using QUIPS—aspects of interrater agreement**. *Diagnostic and Prognostic Research* 2019, **3**(1):5.
